# Accurate and Efficient SAXS/SANS Implementation Including Solvation Layer Effects Suitable for Molecular Simulations

**DOI:** 10.1021/acs.jctc.3c00864

**Published:** 2023-11-03

**Authors:** Federico Ballabio, Cristina Paissoni, Michela Bollati, Matteo de Rosa, Riccardo Capelli, Carlo Camilloni

**Affiliations:** † Dipartimento di Bioscienze, Università degli Studi di Milano, via Celoria 26, 20133 Milano, Italy; ‡ Istituto di Biofisica, Consiglio Nazionale delle Ricerche (IBF-CNR), via Alfonso Corti 12, 20133 Milano, Italy

## Abstract

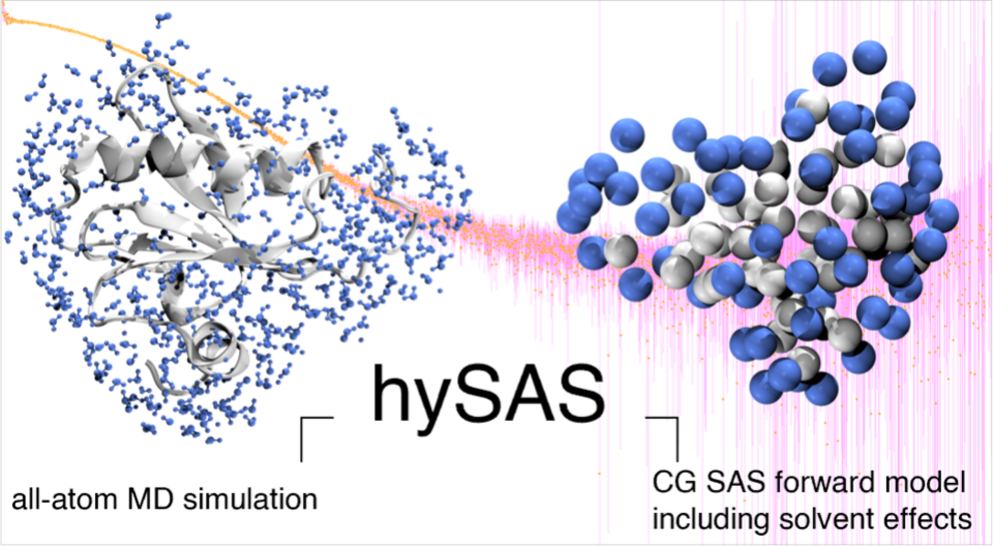

Small-angle X-ray and neutron scattering (SAXS/SANS) provide valuable insights into the structure and dynamics of biomolecules in solution, complementing a wide range of structural techniques, including molecular dynamics simulations. As contrast-based methods, they are sensitive not only to structural properties but also to solvent–solute interactions. Their use in molecular dynamics simulations requires a forward model that should be as fast and accurate as possible. In this work, we demonstrate the feasibility of calculating SAXS and SANS intensities using a coarse-grained representation consisting of one bead per amino acid and three beads per nucleic acid, with form factors that can be corrected on the fly to account for solvation effects at no additional computational cost. By coupling this forward model with molecular dynamics simulations restrained with SAS data, it is possible to determine conformational ensembles or refine the structure and dynamics of proteins and nucleic acids in agreement with the experimental results. To assess the robustness of this approach, we applied it to gelsolin, for which we acquired SAXS data on its closed state, and to a UP1-microRNA complex, for which we used previously collected measurements. Our hybrid-resolution small-angle scattering (hySAS) implementation, being distributed in PLUMED, can be used with atomistic and coarse-grained simulations using diverse restraining strategies.

## 1 Introduction

Small-angle scattering (SAS) techniques based on X-rays (SAXS) or neutrons (SANS) are established, valuable, and widely used tools in structural biology for the characterization of biomolecules in solution. These methods allow the size, shape, stoichiometry, and dynamics of biomolecules to be assessed under near-physiological conditions, using reasonable concentrations, and without the need of labeling agents.^1,2^ Moreover, the size and the disorder level of the system are not a limitation, enabling the study of diverse biomolecular species.^3−5^ Indeed, SAS techniques can efficiently complement nuclear magnetic resonance (NMR) spectroscopy and fluorescence resonance energy transfer (FRET) measurements to provide global features when studying multidomain proteins, intrinsically disordered proteins, and larger complexes.^6^ Furthermore, SAS is particularly suitable for the analysis or the integration with molecular dynamics (MD) simulations, using either reweighing or restraining techniques.^7^ This compatibility arises from the relative simplicity of calculating the forward model from the coordinates of an atomic resolution structure.

Briefly, the SAS intensity of a randomly oriented, *N*—atom, molecule in a vacuum can be calculated by the Debye equation
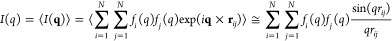
1The intensity is described as a function of the momentum transfer *q* = |**q**| = 4π (sin θ/λ), where 2θ is the scattering angle, λ the source wavelength, and *r*_*ij*_ = |**r**_*ij*_| = |*r*_*i*_ – *r*_*j*_| is the distance from the atom *i* to the atom *j*, which represents the relative position of atoms *i* and *j* in the sample. The notation ⟨···⟩ refers to the spherical average, required to integrate the scattered intensity over all directions that have the same magnitude of *q*. In the case of SAXS, the radiation–matter interaction between the X-ray photon and the electron cloud of atom *i* is described by the atomic scattering factor *f*_*i*_(*q*), which can be approximated with the Cromer–Mann equation
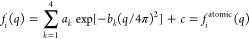
2The empirical and atom-type specific parameters *a*_*k*_, *b*_*k*_, and *c* are available in the International Tables for Crystallography.^8,9^ To account for the solvent effects, each atomic scattering factor *f*_*i*_(*q*) is modified by subtracting a spherical Gaussian which depends on ρ_0_, the electron density of the solvent (e.g., 0.334 e Å^–3^ for bulk water), and ν_*i*_^10^ the volume of the solvent displaced by the atom *i*, following the expression
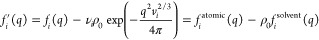
3Since the neutron wavelength is significantly larger than the nucleus dimension, in the case of SANS, the neutron scattering amplitude results to be isotropic, i.e., independent of the scattering angle. Therefore, eq 2 can be approximated to *f*_*i*_^atomic^(*q*) = *b*_*i*_. The *b*_*i*_ constants, which are available in the literature,^11,12^ depend on the number of neutrons and protons that constitute the nucleus. Consequently, isotopes of the same element with different numbers of neutrons, such as hydrogen and deuterium, have different neutron scattering lengths. This feature provides the basis of the contrast variation technique^13^ a powerful advantage of neutron scattering over X-rays, which is usually achieved by using mixtures of hydrogenated and deuterated water in varying proportions. To account for this combination in solvent composition, eq 3 is modified to
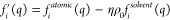
4where
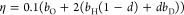
5with *b*_O_, *b*_H_, and *b*_D_ as the neutron scattering amplitudes of oxygen, hydrogen, and deuterium, respectively, and *d* as the deuterium concentration (from 0 to 1, which corresponds to a percentage range of 0–100%). The coefficient 0.1 serves as a scaling factor to account for the 10 electrons per water molecule when converting from electron to molecule density. It should be noted that this modification does not consider the possible effects of hydrogen–deuterium exchange between the solvent and the solvent-exposed residues of the biomolecule.

Although eq 1 accounts for the solvent displaced by the solute in the calculation of the scattering signal, it does not consider the contribution of the solvation shell. This layer depends on solvent–solute interactions and is typically more electron-dense than that of the bulk solvent. For example, the hydration layer has been reported to be up to 20–25% more electron-dense than bulk water.^14−16^ This phenomenon can result in an apparent increase in the radius of gyration of the solute.^17^ The contribution of the solvation layer can be included in calculations through explicit solvent modeling, as implemented in software such as WAXSiS^18,19^ and Capriqorn^20^ or implicitly like in CRYSOL^21^/CRYSON,^14^ FoXS,^22^ and Pepsi-SAXS.^23^ The explicit solvent methods consider the positions of the solvent atoms in the surrounding shell while calculating the scattering signal of the molecule in solution. However, this approach is computationally expensive due to the large number of solvent atoms that must be considered in addition to those of the molecule. Furthermore, it may still be inaccurate because of the limitations of the force field (FF) in the description of water–water and water-solute interactions.^24,25^ Implicit solvent modeling methods, on the other hand, allow the calculation of the solvation layer contribution to the scattering signal without the need to model the solvent atoms explicitly. This reduces the computational cost but results in an approximate representation of the shell.^26^ In general, implicit solvent methods require the introduction of a solvation layer term in eq 3
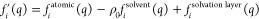
6The way the *f*_*i*_^solvation layer^(*q*) is calculated is slightly different depending on the software used. For example, in CRYSOL 2.x,^27^ this term depends on the contrast between the border layer, which is considered as an envelope of fixed width surrounding the particle, and the bulk solvent.

Equation 1 requires the evaluation of all of the pairwise interatomic distances within the molecule of interest, thus resulting in *N*^2^ calculations, where *N* is the number of the atoms involved, making it highly demanding for large biomolecules. This problem is exacerbated when multiple evaluations of the scattering profile are required, as in the case of MD simulations restrained by SAS data, resulting in severe performance degradation. A successful strategy to mitigate this computational burden is to coarse-grain the representation of the molecule.^28−32^ This simplification can be achieved by combining the scattering behavior of groups of atoms into larger beads while preserving the overall scattering properties of the molecule. This is made possible by the intrinsically low-resolution nature of SAS data, which are more sensitive to the overall shape and size of the molecule rather than its atomic level details. Depending on the specific coarse-graining method used, both the criteria for assigning atoms to beads and the placement of their center can vary significantly. In general, for a coarse-grained system, eq 1 becomes
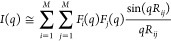
7where *M* is the number of beads, *R*_*ij*_ is the relative distance between the center of the bead *i* and the center of the bead *j*, and *F*(*q*) is the bead form factor, which mirrors the scattering intensities of individual beads. There are several approaches to calculating *F*(*q*); among them, the single-bead approximation (SBA) proposed by Yang et al.^28^ has proved to be one of the most computationally streamlined methods, which is fast but dependable. According to SBA
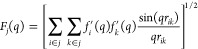
8where *f*_*i*_^′^(*q*) and *f*_*k*_^′^(*q*) are the atomic scattering factors of the atoms *i* and *k* belonging to bead *j*, which are modified to include only the solvent-excluded volume term as in eqs 3 and 4, for SAXS and SANS, respectively. Niebling et al.^32^ have effectively applied the SBA with the Martini 2.2^33^ coarse-grained scheme to derive SAXS bead form factors for proteins, and we have further extended it to nucleic acids.^34^ With this forward model, we used SAXS data to restrain simulations based on the Martini force field^35^ but also based on atomistic force fields, for both proteins and nucleic acids. In this latter hybrid scheme, called the hySAXS approach, the simulation is performed at atomic resolution, while the SAXS intensity of the respective frames is calculated on a coarse-grained model.^36−39^

Even when the Martini representation is used to determine the scattering intensity with hySAXS, the study of relatively large systems can be challenging. Furthermore, this approach was not designed to account for possible corrections due to the solvation layer effects. Here, we present a novel hySAS method for proteins and nucleic acids that is faster and more accurate with the inclusion of a solvent layer contribution and extended to the calculation of SANS intensity. As a case study, we applied the hySAS approach to determine the conformational ensemble of human gelsolin (GSN). This 83 kDa protein (reviewed by Nag et al.^40^) is composed of six homologous domains (named G1 to G6) connected by flexible linker regions and is considered a master regulator of actin dynamics, thanks to its severing and capping activities.^41^ GSN and the other members of the superfamily play an important role in several physiological processes, such as cell division and mobility, trafficking, signal transduction, immunomodulation, and inflammation.^42,43^ GSN is also responsible for a hereditary amyloidosis,^44^ and it is involved in several other diseases, particularly cancer (reviewed by Li et al.^45^). Each GSN domain harbors a Ca^2+^ binding site, and binding to the ion triggers local changes and domain rearrangements that shift the protein from a closed to an open conformation.^46^ In the absence of Ca^2+^, the actin binding sites are buried, limiting the ability of the GSN to interact with actin filaments. In this inactive state, GSN can be crystallized,^47^ but the resolution is relatively low and several stretches of the protein are too flexible to be modeled; such flexibility has been shown to be relevant for GSN physiopathology.^48−50^ In this work, we have determined the ensemble of gelsolin structures in the closed and inactive state using SAXS data measured in the absence of calcium. Furthermore, as a second example of the applicability of hySAS, we refined a previously published protein–RNA complex. This newly introduced hySAS and our previous implementations are already available in the ISDB^51^ module of PLUMED^52,53^ software, an open-source software designed to enhance and extend various MD engines or to be used as a stand-alone package to perform a wide range of advanced analysis of complex biomolecular systems.

## 2 Theory and Methods

### 2.1 SAS Form Factors with the Solvation Layer Contribution

Here, we introduce a novel hySAS method for proteins and nucleic acids where we use a single-bead (1B) representation to describe the scattering behavior of an amino acid and a three-bead (3B) mapping for a nucleotide, one for the phosphate group, one for the pentose sugar, and one for the nitrogenous base. This choice allow us to achieve better performance and to alleviate a source of inaccuracy in the Martini representation, specifically the need to extrapolate the bead form factor when it assumes negative values.^32^ Importantly, to include the solvent layer contribution for small *q* values, we reformulate the SBA *F*(*q*) as the sum of three terms
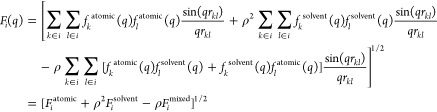
9This approximation allows the *F*_*i*_^atomic^, *F_i_*^solvent^, and *F_i_*^mixed^ terms to be precalculated separately and for each bead type, regardless of the solvent-specific ρ_0_ (and the deuteration fraction in the case of SANS). Therefore, in addition to the option of using a buffer other than bulk water, it is possible to assign modified solvation densities to different beads as a proxy for the effect of the solvation shell. More precisely, the ρ_0_ value of the beads exposed to the solvent can be adjusted to implicitly include the solvation layer contribution (SLC) through a user-defined parameter. This correction can be described as ρ = (ρ_0_ – SLC parameter). For this purpose, the solvent-accessible surface area (SASA) for each amino acid, nucleotide sugar, phosphate group or base is calculated on the fly during the MD simulation or only for a single frame using the efficient LCPO method.^54^ Of note, in the case of SANS, the hydrogen–deuterium exchange is also considered. To achieve this result, we have precalculated the three terms of eq 9 for each bead type using both deuterium *f*^atomic^(*q*) and hydrogen *f*^atomic^(*q*). For the beads exposed to the solvent, each time eq 9 is solved, the terms obtained with deuterium *f*^atomic^(*q*) are used instead of those obtained with hydrogen *f*^atomic^(*q*), with a probability equal to the deuterium concentration in the buffer. For the same bead type, *f*^solvent^(*q*) is identical for SAXS and SANS, both for hydrogenated and deuterated beads, as it depends exclusively on the parameter ν. The ρ_0_ value, the SLC parameter, the SASA threshold to consider a residue solvated, and the deuterium fraction in SANS can be defined by the user.

### 2.2 Bead Form Factor Parametrization and Validation

We computed the *F*_*i*_^atomic^, *F*_*i*_^solvent^, and *F*_*i*_^mixed^ terms of eq 9 for all of the amino acids, as well as for nucleic acid bases, the pentose sugars, and the phosphate group, for both SAXS and SANS. Concerning proteins, the three terms per amino acid were calculated and averaged over 1000 frames extracted at equidistant intervals from a 2.7 μs MD trajectory of GSN. The heterogeneous structural composition of this 755-residue protein makes it an ideal model for a comprehensive conformational sampling. Indeed, in addition to encompassing all of the standard amino acids, GSN features an IDP-like N-terminal region of ∼25 residues and six structured domains rich in α-helices and β-sheets, connected by flexible linkers. To validate the transferability of the GSN terms to other systems, we generated two additional independent term sets, derived from a 270 ns MD simulation of the B1 immunoglobulin-binding domain of streptococcal protein G (B1), and from a 1.35 μs trajectory of the green fluorescent protein (GFP). The three components of the bead form factors of all of the standard amino acids have been calculated. Furthermore, we have also included the scattering behavior of the histidine with both the δ- and ε-nitrogen of the imidazole ring protonated. A different strategy was employed for nucleotides. Considering the lower accuracy of the FF for nucleic acids,^55^ we preferred to calculate and average the terms from nonredundant molecular structures obtained from the Protein Data Bank (PDB). We used a set of 167 noncomplexed DNA structures^56^ and a set of 75 RNA structures^57^ that we had already prepared and used in our previous work.^34^ To validate the parameters, 120 DNA and 43 RNA structures with no missing heavy atoms were selected from these repositories as the training subset, while the remaining structures were used as the validation subset. The final terms were computed on the two complete repositories (Table S1). We derived the form factor components of the five nucleobases (adenine, cytosine, thymine, uracil, and guanine), the phosphate group, and the DNA and RNA pentose sugars. In addition, we included two other DNA/RNA bead types for the 5′-end and the 3′-end pentose sugar with a hydroxyl moiety at carbon C5′ and C3′, respectively. Finally, each term belonging to either a protein or nucleic acid was fitted to a sixth-order polynomial. This means that *F*_*i*_^atomic^, *F*_*i*_^solvent^, and *F*_*i*_^mixed^ are described by a total of 21 parameters.

### 2.3 Computational Details

Protein bead form factor parametrization was performed on mature human GSN. The initial model was determined from the PDB entry 3FFN, whose missing loops and N-terminus were reconstructed using AlphaFold2.^58^ Regarding the 56 residues B1, and the 230 residues GFP, the structures are based on PDB entries 1PGB and 1GFL, respectively. All of the structures were prepared with the following procedure. The histidine orientation and protonation states were optimized using Schrödinger Maestro Suite, release 2021-4.^59^ The topology was built using DES-Amber^59^ FF and the system was solvated with the TIP4P-D^60^ water model in a dodecahedron box with a NaCl concentration of 100 mM. After two preliminary minimization steps (steepest descent and conjugate gradient algorithms), a 2 ns long NPT simulation was performed with the protein atoms restrained to their minimized positions. For GSN, 675 ns of classical MD simulation was computed for each of the 4 replicas, collecting a total of 2.7 μs. For GFP, we ran a single replica MD simulation of 1.35 μs, while for B1, we ran 4 replicas for 67.5 ns each, reaching 270 ns.

The plain MD simulations of GSN, B1, and GFP were also used to evaluate the performance and accuracy of calculating SAS intensities at different resolutions. For nucleic acids, we followed the previous procedure to prepare and perform a 35 ns simulation of the 1,187 nucleotides large subunit ribosome fragment (PDB ID 1Z58) and a 14 ns simulation of single-stranded 12-mer RNA (AGUAGAUUAGCA). The former was used to assess the timing of the SAS intensity calculation and the latter to assess the accuracy.

For the GSN refinement, driven by SAXS-restrained MD simulation, the previous structure was modified. Since the experimental SAXS measurements were collected on a full-length GSN fused to a N-terminal His_6_-tag, we modeled an additional 23 residues, corresponding to the sequence “MGSSHHHHHHSSGLVPRGSHMAS”, resulting in a 778-residue protein that was prepared as described previously. We ran 2x 1 μs metainference^61^ multireplica simulations (10 replicas, 100 ns each), one with and one without the solvation layer correction enabled. The representative SAXS intensities selected as restraints range between the *q* values of 0.01 Å^–1^ and 0.25 Å^–1^ with a stride of 0.015 Å^–1^. The analysis was performed over the last 50 ns of each trajectory.

Regarding the protein–RNA complex refinement, we adopted the MD input files prepared in our previous work.^34^ In summary, AMBER14SB^62^ FF with parmbsc1^63^ parameters and the TIP3P^64^ water model were used to build the topology. To preserve the protein–RNA interface, we introduce harmonic biases on the distances between the phenylalanine residues and bases involved in nonbonded interactions; furthermore, we also added a restraining potential on the secondary structures of the protein, following the same procedure described by Kooshapur et al.^65^ The metainference simulations were performed for 4.5 ns with and without the solvation layer correction activated, using 35 selected SAXS intensities with *q* values between 0.008 Å^–1^ and 0.3 Å^–1^ as restraints.

All of the simulations were performed using GROMACS 2021.6,^66^ PLUMED2,^52,53^ and the PLUMED-ISDB^51^ module. Plots were generated using the matplotlib^67^ 3.6.0 package, while the open-source software VMD^68^ and PyMOL^69^ were used for structural visualization of biomolecules. Relevant input files and trajectories are available on Zenodo^70^ and the PLUMED-NEST as plumID:23.029.

### 2.4 Gelsolin Expression, Purification, and SAXS Data Collection

Recombinant full-length GSN protein, carrying an N-terminal His_6_-tag, was produced as previously described.^50,71^ Briefly, the human plasma isoform of GSN devoid of the signal peptide (mature form) was produced in *Escherichia coli* SHuffle cells (New England Biolabs) upon addition of 0.5 mM IPTG and incubation for 16 h at 18 °C. Cells were lysed in a Basic Z Bench top (Constant Systems Limited, U.K.) at 25 kPSI, and the clarified extract passed through a HisTrap HP column (all chromatographic media from GE-Healthcare). Further polishing was obtained by anion exchange (Resource Q), followed by size-exclusion chromatography (HiLoad 16/600 Superdex 200). For SAXS analysis, the protein was diluted to 2.01 mg/mL in 20 mM HEPES, pH 7.4, 100 mM NaCl, and 1 mM EDTA. GSN batch data were collected at the B21 BioSAXS beamline of the Diamond Synchrotron (Didcot, Oxfordshire, UK).^72^ Data and model are deposited in the SASBDB^73^ as SASDSN7.

## 3 Results and Discussion

### 3.1 Single-Bead Mapping for Amino Acids and Three-Bead Mapping for Nucleotides Are Fast and Accurate for Small *q* Values

**Figure 1:**
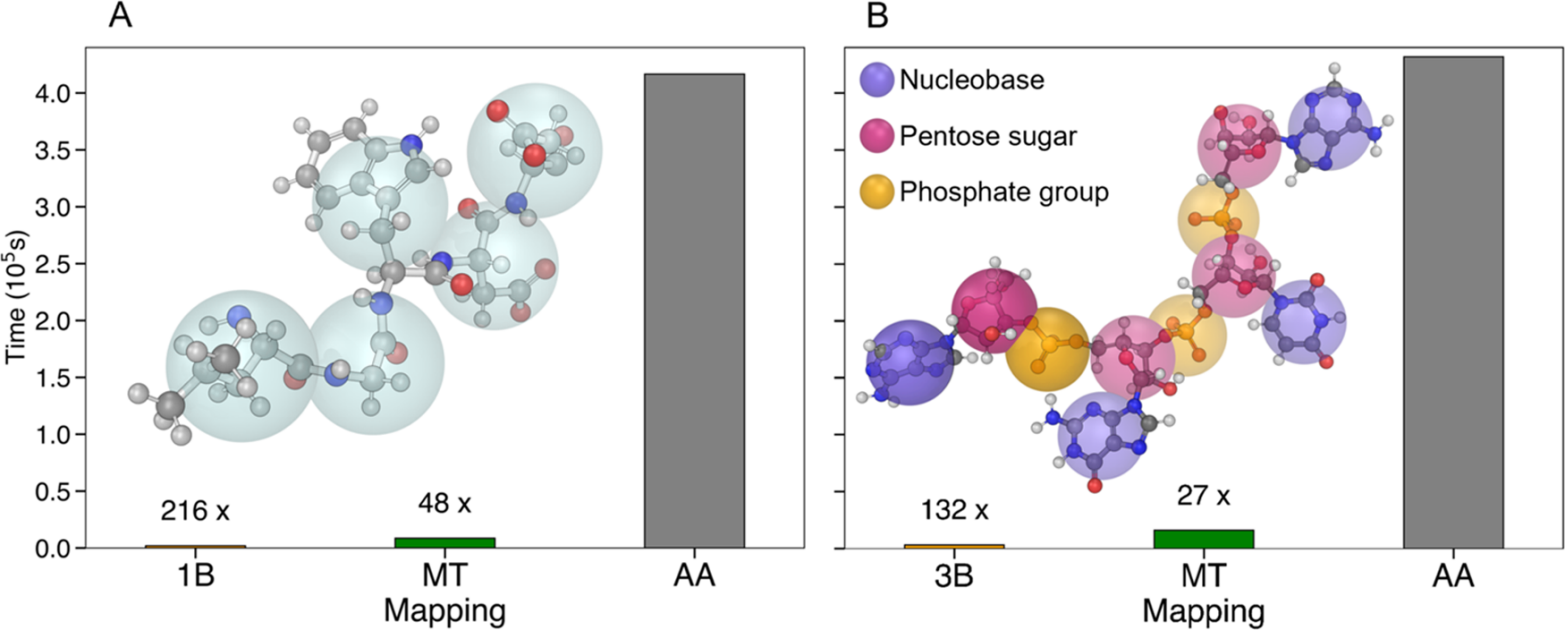
SAS intensity calculation timings. (A) The 6442 frame MD trajectory of 755 residues GSN was used as input for PLUMED-ISDB plugin to calculate the corresponding SAS intensities for 31 *q* values, using different mapping resolutions. The time required for completion at AA details (11,558 atoms) is 416,594 s and with MT mapping (1627 beads) is 8,615 s, while for 1B (755 beads) is 1925 s. 1B and MT are 216 times and 48 times faster than AA, respectively. As an example, five residues are shown at atomistic (ball and sticks visualization) and 1B resolution (light blue beads). (B) The 500 frames MD trajectory of 1187 nucleotide RNA strand was used to calculate the corresponding SAS intensities for 31 *q* values. The time required for completion at AA resolution (38,287 atoms) is 432,022 s and for MT mapping (7796 beads) is 15,912 s, while for 3B (3,560 beads) is 3263 s. 3B and MT are 132 times and 27 times faster than AA, respectively. As an example, four nucleotides are shown at atomistic (ball and sticks visualization) and 3B resolution (nucleobase in blue, pentose sugar in violet, phosphate group in orange). The timings were evaluated under the same conditions on a single core of a workstation equipped with an Intel Xeon E5-2660v3 CPU.

To assess the impact of the number of elements in a system on the speed of the SAS intensity calculation, we compared the time required by PLUMED to determine intensities from MD trajectory frames. We used different resolutions, including all-atom (AA), Martini scheme with transferable parameters (MT), and single-bead per amino acid (1B)/three beads per nucleotide (3B) mappings with the corresponding transferable parameters. For the analysis, we selected a GSN trajectory consisting of 6442 frames to evaluate the performance on proteins and a 500 frames trajectory of a large subunit ribosome fragment to evaluate nucleic acids. The intensities were calculated for 31 *q* values, in the range 1 × 10^–10^ to 0.3 Å^–1^, every 0.01 Å^–1^. As expected, the resolution had a dramatic effect on the calculation time. For proteins, it took approximately 5 days to determine the SAS intensity at AA details (11,558 atoms). The same calculation was achieved in about 143 min (48.4-fold speedup) using the MT mapping (1627 beads) and approximately 32 min (216.4-fold speedup) using the 1B representation (755 beads) (Figure 1A). Similarly, for nucleic acids, the calculation time reduced from around 5 days at AA resolution (38,287 atoms) to about 265 min (27.2-fold speedup) using MT mapping (7796 beads) and about 54 min (132.4-fold speedup) using the 3B representation (3560 beads) (Figure 1B).

**Figure 2:**
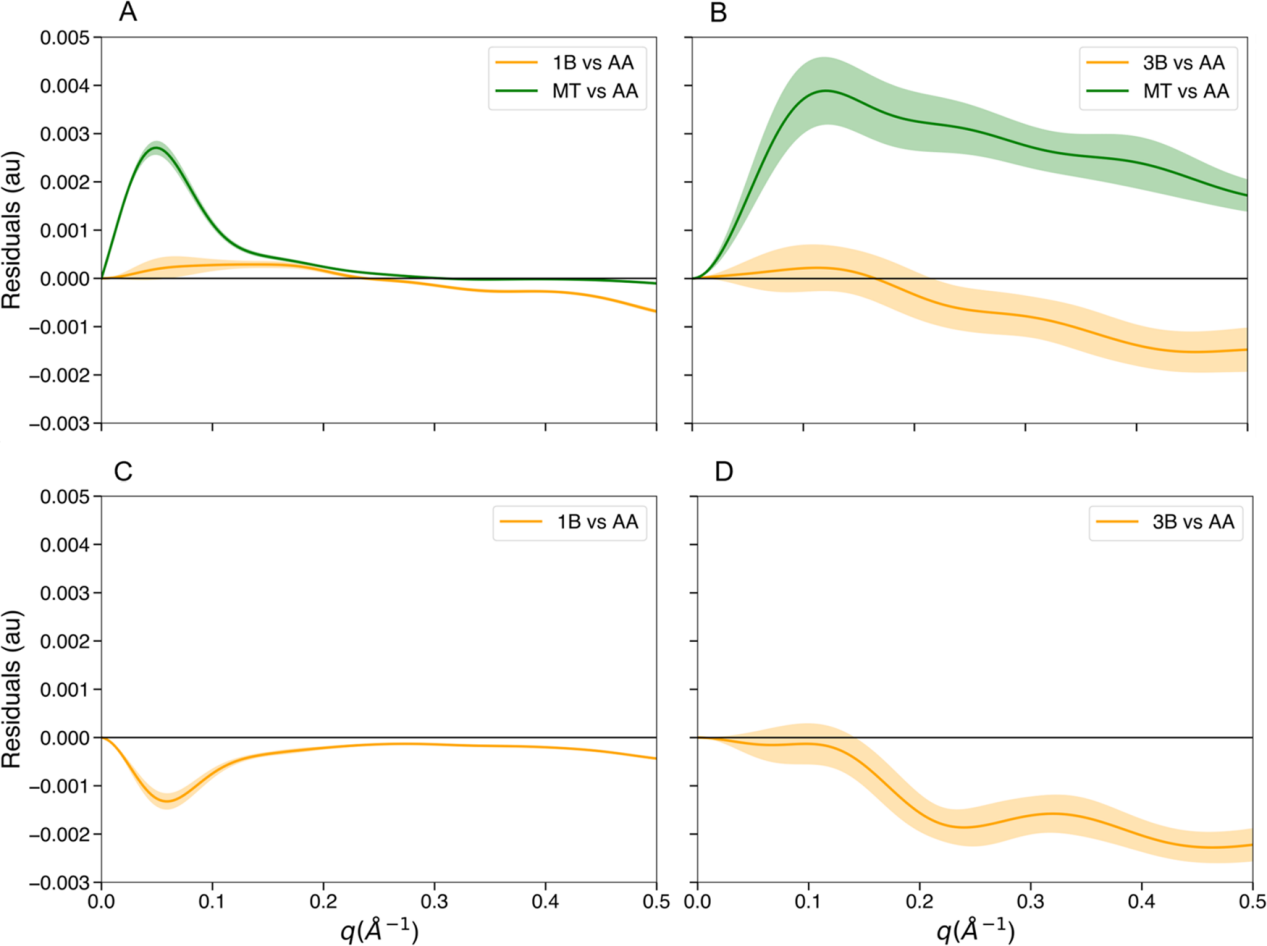
Validation of the 1B/3B mappings in the calculation of scattering intensities. The SAS profile of each frame from MD trajectories was calculated at atomic and coarse-grained resolution, for 201 *q* values ranging from 1 × 10^–10^ to 0.5 Å^–1^. (A) Average and standard deviation on 6442 GSN frames of the SAXS residuals between MT and AA (green) and between 1B and AA (orange). (B) Average and standard deviation on 7256 12-mer RNA frames of the SAXS residuals between MT and AA (green) and between 3B and AA (orange). (C) Average and standard deviation on 6442 GSN frames of the SANS residuals between 1B and AA (orange). (D) Average and standard deviation on 7256 12-mer RNA frames of the SANS residuals between 3B and AA (orange).

In addition to performance evaluation, we also assessed the accuracy of 1*B*/3B mappings and parameters in the calculation of scattering intensities. For this benchmark, we selected 6442 equidistant frames from the GSN MD simulation, 6502 from B1, 9622 from GFP, and 7256 from the 12-mer RNA strand and calculated the SAXS and SANS intensity in both coarse-grained and atomistic details for 201 *q* values, in the range of 1 × 10^–10^ to 0.5 Å^–1^, every 0.0025 Å^–1^. For each frame and each *q* value, the intensity calculated with 1*B*/3B was compared with the corresponding intensity at atomistic resolution, which was taken as the reference. For SAXS we also included the comparison between MT and AA. The GSN SAXS intensities calculated with 1B mapping showed better agreement with those obtained with AA resolution than with MT up to 0.3 Å^–1^, since the difference (residuals) between 1B and AA intensities is smaller than the difference between MT and AA for the same set of *q* values (Figure 2A). A similar behavior has been observed also for B1 (Figure S1A, left panel) and GFP (Figure S1B, left panel) SAXS intensities. This phenomenon, which is probably amplified by the approximation introduced in the calculation of the MT bead form factors, shows that for small *q* values, the atomic details are not critical in the determination of the intensity. Considering B1, which is the worst case scenario we observed, the SAXS intensity computed with 1B differs by less than 0.5% from that calculated at atomistic resolution in the range 0–0.3 Å^–1^. Regarding the SANS intensity calculation with 1B mapping, the results obtained for GSN (Figure 2C) and GFP (Figure S1B, right panel) were comparable to those of SAXS, whereas for B1, the accuracy decreased, with a maximum difference between 1B and AA scattering profiles of about 1.5% (Figure S1A, right panel). As for the proteins, the calculation of the SAXS intensity on RNA with 3B mapping also proves to be accurate, with better agreement with AA resolution than with MT (Figure 2B). Finally, the difference between the RNA SANS intensity computed with 3B and that computed with AA shows a level of accuracy close to that observed for SAXS (Figure 2D). These results were obtained without considering the solvation layer contribution. To assess the transferability and validate the 1B parameters obtained from GSN, we generated additional independent sets of parameters from B1 and GFP. Using the 1B parameters obtained from B1, we calculated the SAXS intensities on the B1 trajectory frames and compared them with the corresponding AA intensities. The same B1 frames were employed to calculate the 1B intensity using the parameters obtained from GSN, and these intensities were also compared with the AA profiles. The two obtained residuals are nearly superimposable (Figure S2A), differing from each other by less than 0.1% at most. We followed the same procedure with GFP, and similarly, the residuals calculated with the 1B parameters from GFP are in strong agreement with the residuals calculated with the 1B parameters from GSN (Figure S2B). To validate the 3B parameters, the nucleic acid repositories previously described in Section 2 were divided into two subsets. We selected 120 DNA and 43 RNA PDB files as the training set to compute the 3B form factor parameters since all of the heavy atoms are solved in these structures. We calculated the SAXS intensity of each structure belonging to this set with 3B mapping and at AA resolution and evaluated the respective residuals. We performed the same analysis using the 3B parameters obtained from the training set on the remaining 47 DNA and 32 RNA structures, which we considered as the validation subset. The average of the residuals from the training set and the average of the residuals from the validation set differed by a maximum of 0.12%. Furthermore, although the residuals from the validation set are more dispersed, the average is closer to the reference than the average of the residuals from the training set (Figure S3).

### 3.2 Inclusion of the Solvation Layer Contribution Allows Matching the SAXS Intensity Calculated by WAXSiS

**Figure 3:**
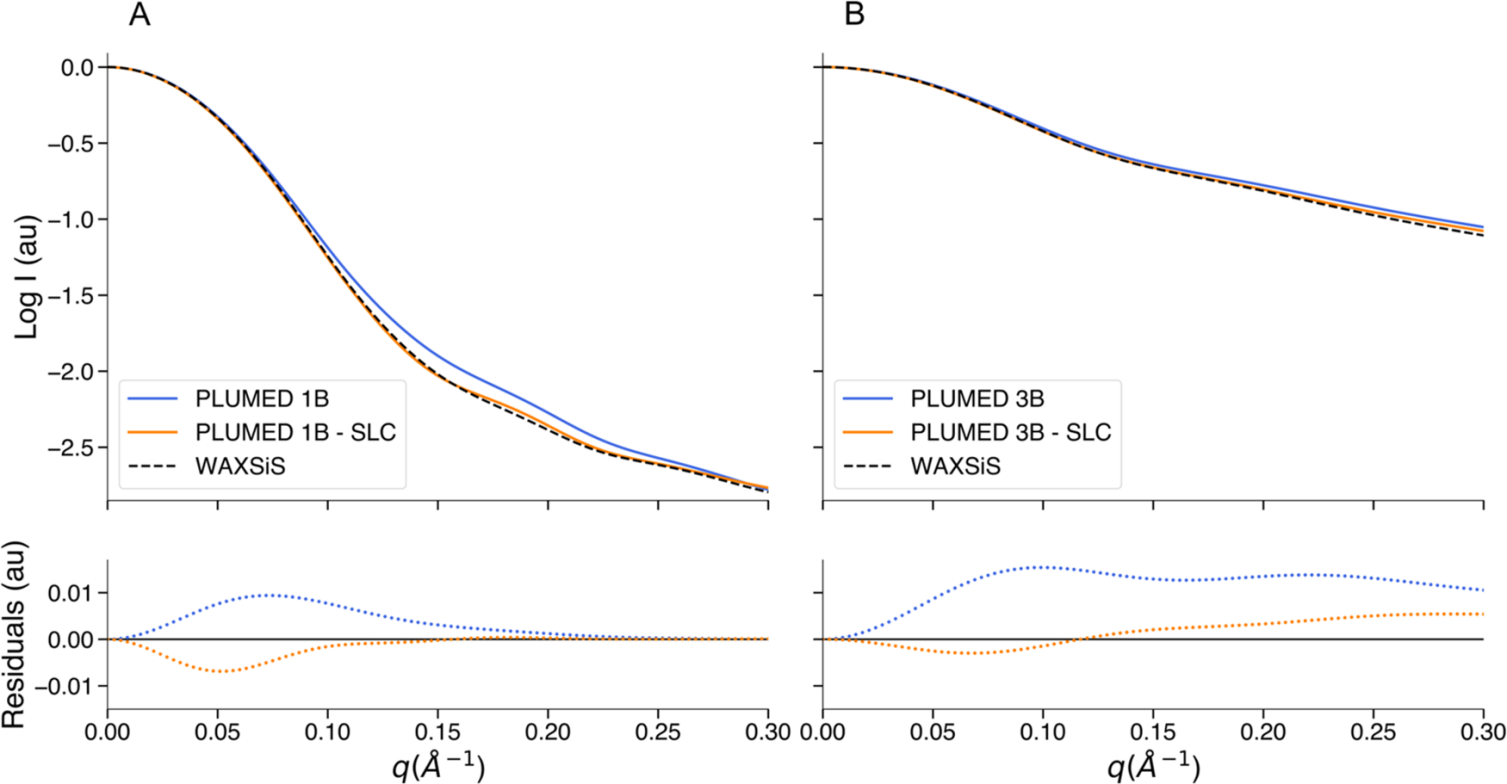
Solvation layer contribution in the 1B/3B SAXS intensity calculation. (A) Upper panel: logarithm of the SAXS profile of a representative, randomly selected, GSN frame calculated using 1B mapping (blue), 1B mapping with the best combination of SLC (0.08) and SC (0.6 nm^2^) found for this frame (orange), and using WAXSiS (black). Bottom panel: residuals of 1B (blue) and 1B with SLC (orange) using the WAXSiS intensity as the reference. (B) Upper panel: logarithm of the SAXS profile of a representative, randomly selected, 12-mer RNA frame calculated using 3B mapping (blue), 3B mapping with the best combination of SLC (0.120) and SC (1.0 nm^2^) found for this frame (orange), and using WAXSiS (black). Bottom panel: residuals of 3B (blue) and 3B with SLC (orange) using the WAXSiS intensity as the reference. All of the SAXS intensities were calculated for 101 *q* values, up to 0.3 Å^–1^.

The 1B and 3B form factors can be modified to include the solvation layer contribution in the SAS intensity calculation. Whether for a single PDB file or an MD trajectory, this process requires the calculation of the SASA to assess which beads are exposed to the solvent. This procedure is performed by the LCPO^54^ algorithm implemented^74^ in PLUMED. The reliability of the method was verified by comparing the results obtained with LCPO with the results obtained for the same frames with the sasa module^75^ integrated in GROMACS (Figure S4). To evaluate the SLC, we used as a reference the intensities calculated by WAXSiS (Wide Angle X-ray Scattering in Solvent), a web server hosted at Saarland University, which allows the calculation of SAXS/WAXS profiles based on short MD simulations in an explicit solvent.^18,19^ We extracted 10 equidistant frames from each of the previously described trajectories of GSN, B1, GFP, and 12-mer RNA. For all of these frames, we calculated the SAXS intensity using 1B/3B mapping with the SLC parameter set to 0.04, 0.06, 0.07, 0.08, 0.09, 0.095, 0.10, 0.11, and 0.12 and with the SASA cutoff (SC) of 0.4, 0.6, 0.7, 0.8, 1.0, and 1.2 nm^2^, in all of the possible combinations. The same frames were used as input to calculate the SAXS profiles with WAXSiS. We specified in the web server options an explicit solvent envelope of 7 Å from the surface of the biomolecule, and we selected the maximum available simulation length (2 × 10^6^/*N*^–0.77^ frames, where *N* is the approximate number of atoms in the hydration layer). As in the previous analyses, we calculated the residuals between the intensity computed with 1B/3B and the intensity computed with WAXSiS, that we consider as the reference. Although some combinations of SLC and SC gave surprising results, leading to SAXS profiles practically identical to those calculated by WAXSiS (Figures 3 and S5), we found that using any of the indicated values of SLC and SC gave a better agreement with WAXSiS intensity than using 1B/3B without SLC. For a clearer overview, we computed the root-mean-square error (RMSE) between the logarithm (base 10) of the SAXS intensity calculated with AA, MT, and 1B/3B (with all of the SLC/SC combinations) and the logarithm of the SAXS intensity calculated with WAXSiS. The results obtained from all of the extracted frames were averaged for each system (Table S2). This analysis showed that the 1B/3B with SLC gave better results than 1B/3B without SLC but also compared to MT and AA resolution. For GSN, B1, and GFP, the SLC values that lead to the best results are generally between 0.08 and 0.1 with the SC of 0.7–0.8 nm^2^. Instead, for the 12-mer RNA, an SLC greater than 0.1 with SC between 0.8 and 1 nm^2^ is more in agreement with the reference.

### 3.3 Solvation Layer Contribution Results in a Smaller Radius of Gyration and an Overall Decrease in the Fluctuations of Gelsolin

**Figure 4:**
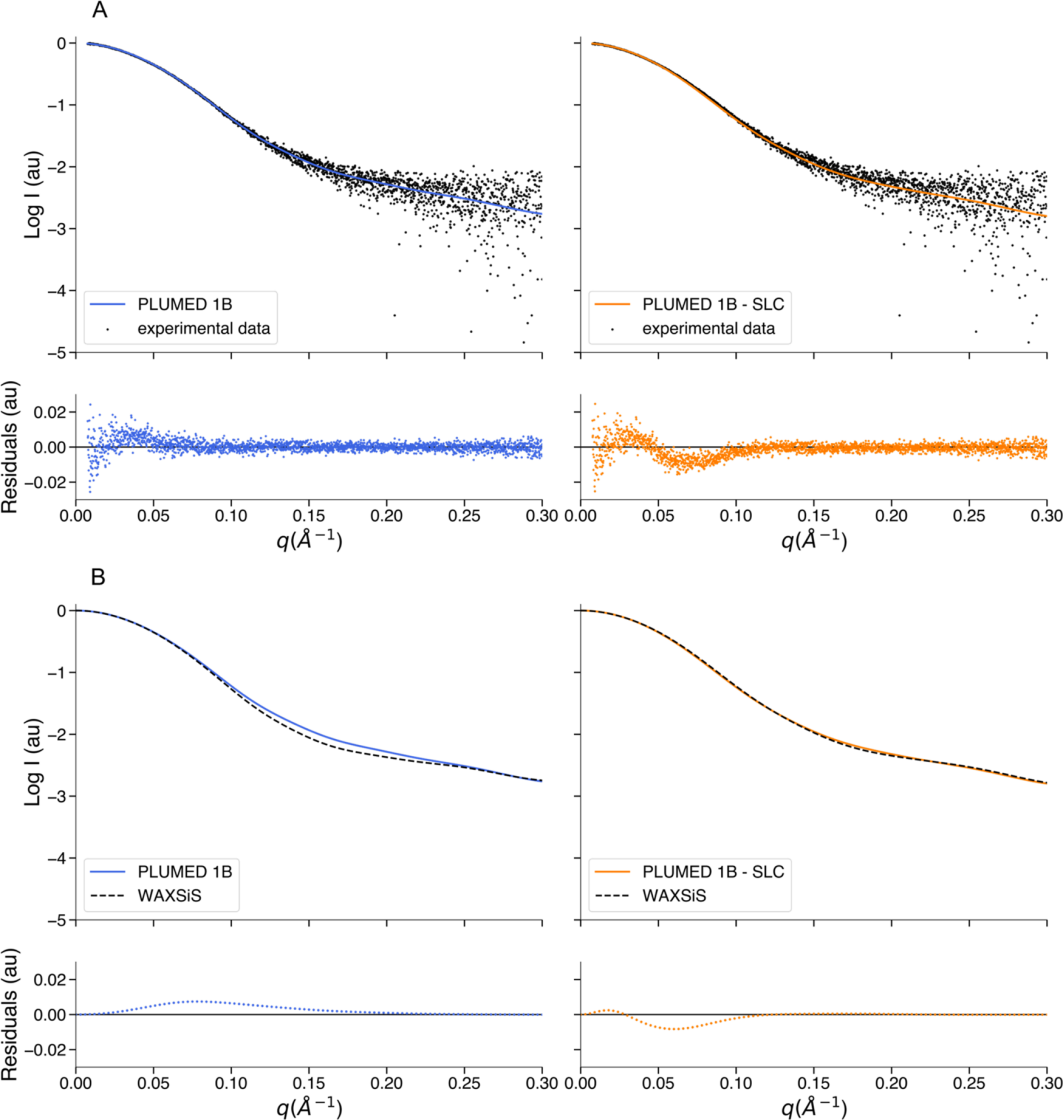
Agreement between hySAS, experimental SAXS data, and WAXSiS for the gelsolin ensembles. (A) Left panel: comparison between the logarithm of the average GSN SAXS profile calculated using 1B mapping without SLC (blue) and the logarithm of the experimental SAXS data (black dots). Right panel: comparison between the logarithm of the average GSN SAXS profile calculated using 1B mapping with SLC (orange) and the logarithm of the experimental SAXS data (black dots). (B) Left panel: comparison between the logarithm of the average GSN SAXS profile calculated using 1B mapping without SLC (blue) and the logarithm of the average WAXSiS profile (black dashed line). Right panel: comparison between the logarithm of the average GSN SAXS profile calculated using 1B mapping with SLC (orange) and the logarithm of the average WAXSiS profile (black dashed line). All of the residuals are calculated as the difference between the two intensities considered.

**Figure 5:**
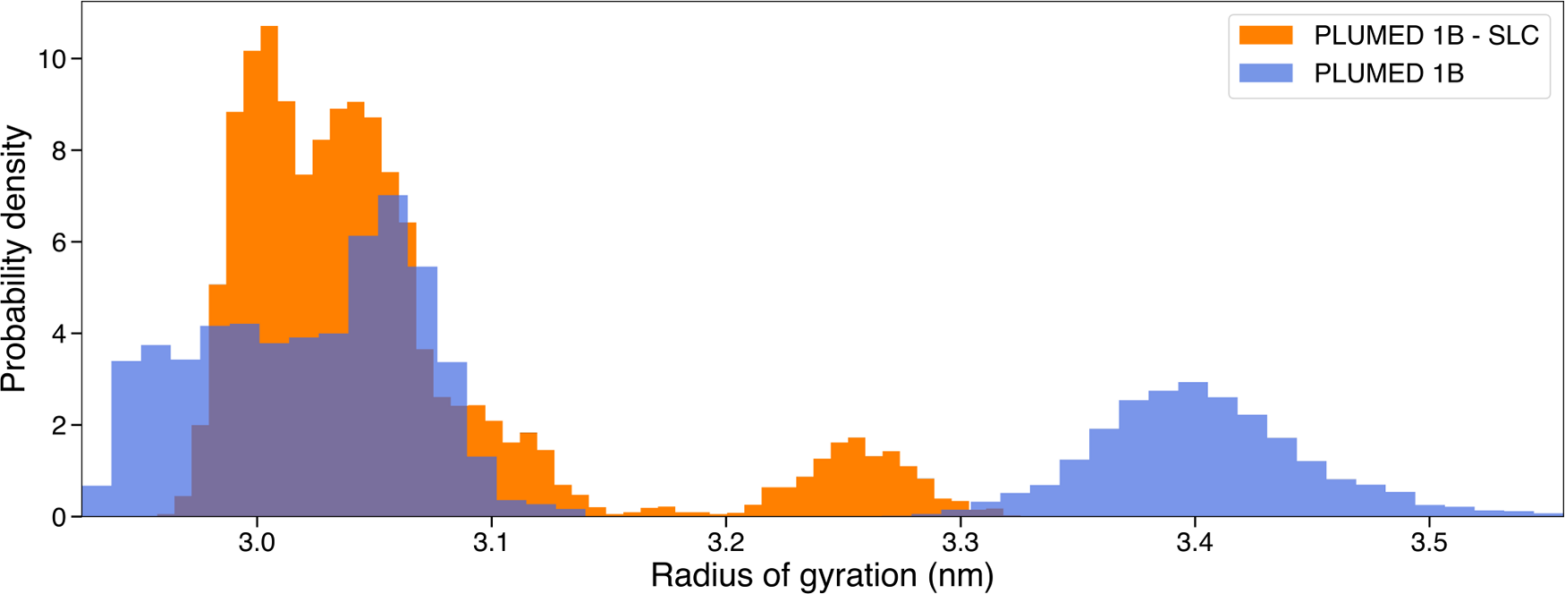
Radius of gyration and probability density histograms of GSN ensembles. The probability density distribution of the radius of gyration was calculated over 10,000 frames obtained using hySAS with SLC, colored in orange, while the distribution calculated over 10,000 frames obtained using hySAS without SLC is colored in blue. The area under each histogram integrates to 1.

**Figure 6:**
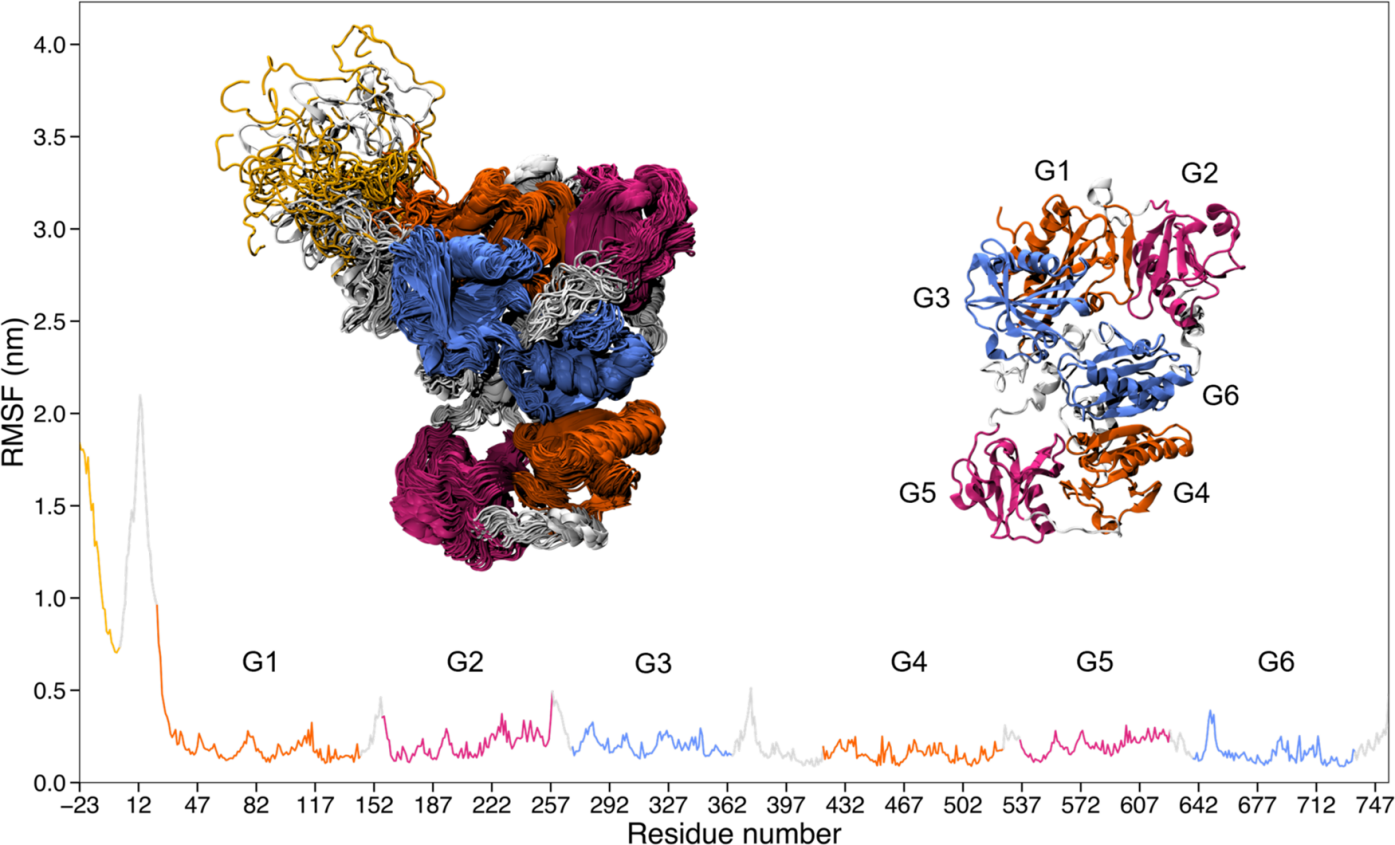
RMSF analysis of the GSN ensemble (with SLC). The flexibility of the protein was assessed by calculating the root-mean-square fluctuation of all residues. The residue numbering sequence on the *x*-axis includes the N-terminal His_6_-tag (from −23 to −1) and the full-length human plasma isoform of GSN (1 to 755). The domains sharing the highest sequence and structural similarity are shown with the same color code: G1 and G4 are colored in orange, G2 and G5 in purple, and G3 and G6 in blue. The linkers and tails are colored in light gray, while the His_6_-tag is colored in yellow. On the left, 50 equidistant frames from the analyzed trajectories are superimposed as a representative example of the conformational ensemble. The GSN structure on the right is that obtained by X-ray crystallography (PDB ID: 3FFN).

The size and large structural variability of GSN made it an excellent candidate to provide a realistic evaluation of our method and to assess its applicability to practical scenarios. Specifically, we generated two independent GSN conformational ensembles through metainference multireplica simulations, using experimental SAXS data as a restraint and the 1B mapping and parameters to compute the forward models. One of the two ensembles was obtained by enabling SLC with a value of 0.08 and with the SC of 0.7 nm^2^. We selected these settings based on the result of the analyses reported in the previous paragraph, as well as being particularly appropriate for GSN, this combination was also reasonable for B1 and GFP (Table S2). To define the ensembles, we considered only the second half of the trajectory of each replica, where the correlation between the forward model and the experimental intensity was stably close to 1, with a constant metainference score. From each ensemble, we extracted 1,000 equally distant frames, recalculated the SAXS profile using PLUMED with 1B mapping, and determined the average profile. For the ensemble frames with hydration layer correction, we used the same SLC and SC settings as those for the refinement. The two average profiles, representing the two ensembles, were directly compared with the experimental SAXS data (Figure 4A). We observed that both profiles show good agreement with the experimental SAXS data, with a chi-squared of 0.8 and 1.4 without and with the SLC term, respectively. To verify that our hydration layer correction is working properly, we also calculated the SAXS intensities using the WAXSiS web server and determined the corresponding average profiles. We compared the PLUMED profile with the WAXSiS profile of the ensemble obtained without SLC (Figure 4B, left panel) and the PLUMED profile with the WAXSiS profile of the ensemble obtained with SLC (Figure 4B, right panel). In this case, we found that the agreement between PLUMED and WAXSiS is higher when comparing the intensities calculated from the ensemble with SLC (RMSE: 1.7 × 10^–2^) than when comparing the intensities calculated from the ensemble without SLC (RMSE: 6.4 × 10^–2^). This indicates that although the hydration layer contribution in the SAXS intensity calculation is not critical to match the experimental data, hySAS can match WAXSiS with the appropriate SLC/SC settings. We analyzed both the ensembles in terms of radius of gyration and root-mean-square fluctuations (RMSFs) to gauge the effect of the SLC on the resulting conformations. Although both the ensembles showed a bimodal distribution of the radius of gyration, the one obtained with the inclusion of the SLC was, as possibly expected, more compact with an average radius of gyration of 3.05 nm, compared to the one generated without the SLC, which showed an average radius of gyration of 3.14 nm (Figure 5). Interestingly, a similar behavior was observed regarding the RMSF. The ensemble calculated applying the SLC shows systematically lower fluctuations, with an average RMSF of 0.26 nm, compared to the other ensemble, which has an RMSF of 0.38 nm (Figures 6, S6, and S7). Focusing on the SLC-corrected ensemble, the main contribution to the radius of gyration and the RMSF comes from the long N-terminal disordered region with significant fluctuations also found in the two main linkers connecting the G2 domain to the G3 domain and the G3 domain to the G4 domain (Figure 6). Referring to high-resolution data for some of the isolated domains (also in the presence of Ca^2+^ and/or actin),^76−78^ GSN appears reasonably stable, suggesting that the model with smaller fluctuations is preferable.

### 3.4 Solvation Layer Contribution Results in a Lower Radius of Gyration in the Refinement of a Protein–RNA Complex

**Figure 7:**
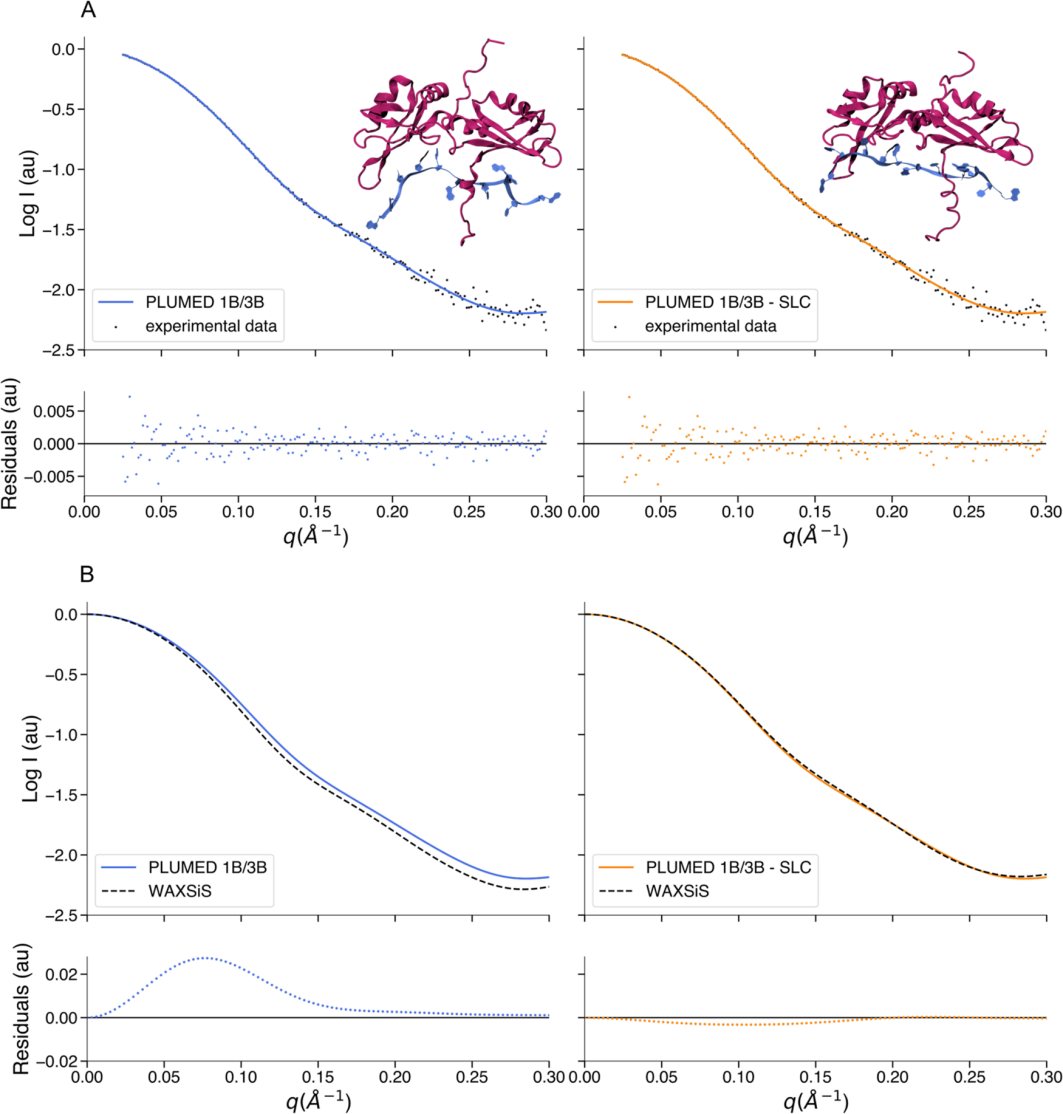
Comparison between hySAS, experimental SAXS data, and WAXSiS for UP1-RNA complex models. (A) Left panel: comparison between the logarithm of the protein–RNA complex SAXS intensity calculated using 1B/3B mapping without SLC (blue) and the logarithm of the experimental SAXS data (black dots). Right panel: comparison between the logarithm of the protein–RNA complex SAXS intensity calculated using 1B/3B mapping with the SLC (orange) and the logarithm of the experimental SAXS data (black dots). The upper right section of each panel shows the protein–RNA complex frame responsible for the relative intensity profile (purple/cartoon representation for UP1, blue/ribbon representation for 12-mer RNA). (B) Left panel: comparison between the logarithm of the protein–RNA complex SAXS intensity calculated using 1B/3B mapping without SLC (blue) and the logarithm of the WAXSiS profile (black dashed line). Right panel: comparison between the logarithm of the protein–RNA complex SAXS intensity calculated using 1B/3B mapping with the SLC (orange) and the logarithm of the WAXSiS profile (black dashed line). All of the residuals are calculated as the difference between the two intensities considered.

In addition to generating conformational ensembles, hySAS can also be used to refine single structures to enhance consistency with experimental SAS data. As an example of the latter application, we choose to improve a model of a previously published protein–RNA complex.^65^ This system consists of the 199-residue unwinding protein 1 (UP1) interacting with a 12-mer single strand derived from the primary transcript of the 18*a* microRNA. The complex was originally refined using metainference, SAXS, and NMR data as restraints and successively tested with hySAXS and the Martini bead form factors.^34^ Here, we repeated the latter test using the same input files and data but 1B and 3B mapping to compute the forward models. We generated a short trajectory with and without SLC with a value of 0.12 and an SC of 0.8 nm^2^. From each trajectory, we obtained a refined structure with a chi-squared of 1 with respect to the SAXS data (Figure 7A). As for the GSN, to verify our method, we compared the intensities computed from the two selected conformations with the corresponding intensities recalculated with the WAXSiS web server. We obtained an RMSE of 6.4 × 10^–2^ between the PLUMED and the WAXSiS logarithm of the intensities (Figure 7B, left panel) when using the conformation generated without employing the hydration layer correction. However, when using the conformation calculated with the SLC, the RMSE drops to 1.2 × 10^–2^ (Figure 6B, right panel). Therefore, also in this case, the use of our SL allows us to obtain SAS profiles in agreement with WAXSiS.

Comparing the two resulting refined structures, it is possible to observe a difference in their radius of gyration, with the one obtained without using the SLC characterized by a radius of 2.26 nm as observed in our previous work,^65^ and the one obtained using the SLC term by a radius of 2.20 nm. This difference is the result of more relaxed terminal regions of UP1.

## 4 Conclusions

The integration of experimental data in simulations is a powerful approach to increase the resolution of the former and the accuracy of the latter.^79−81^ This integration is based on two elements: (i) a forward model for the calculation of an experimental observable, given a conformation and (ii) an integration strategy (e.g., restraints or reweighting based on either the maximum entropy principle or Bayesian inference^82,83^). The forward model should be accurate and computationally efficient when the goal is to apply a restraint in a simulation. In this work, we have presented an implementation of a SAXS and SANS forward model that efficiently exploits the limited resolution of these experimental techniques. In particular, it allows protein and nucleic acid scattering to be represented by a single-bead per amino acid and a three-bead per nucleic acid residue, and more importantly, it enables the effective on-the-fly inclusion of solute–solvent scattering corrections at no cost. We showed that the inclusion of this correction modifies the resulting conformations by mildly decreasing their radius of gyration, as expected, and matching WAXSiS, a more accurate but expensive forward model. The method presented here is already deployed in PLUMED, thus allowing its use in combination with different molecular dynamics engines, restraining strategies including metainference^61^ and maximum entropy^84,85^/caliber^86^ approaches or enhanced sampling techniques such as metadynamics^87^ and umbrella sampling.^88^

## Supporting Information Available

Extensive comparison of the performance of MT, 1B, and AA representation for proteins (considering also SLC), as well as the same comparison for 3B and AA for nucleic acids; table containing the list of PDB accession codes used to compute the 3B parameters; tables with the combinations of SASA threshold and the solvent density for SLC correction for both protein and nucleic acids; RMSF plot for the GSN without SLC; and ΔRMSF plot for the two simulations with and without SLC (PDF)

## Accession Codes

All data are available via Zenodo with record 8192455, the PLUMED-NEST as plumID:23.029, and the SASBDB with accession SASDSN7.

## Abbreviations

SAXS: small-angle X-ray scattering
SANS: small-angle neutron scattering
MD: molecular dynamics
SLC: solvation layer contribution
GSN: gelsolin
B1: B1 immunoglobulin-binding domain of streptococcal protein G
GFP: green fluorescent protein
FF: force field
PDB: protein data bank
RMSE: root-mean-square error
RMSF: root-mean-square fluctuation

## References

[ref1] Tuukkanen A. T.; Spilotros A.; Svergun D. I. Progress in small-angle scattering from biological solutions at high-brilliance synchrotrons. IUCrJ. 2017, 4 (5), 518–528. 10.1107/S2052252517008740. 28989709 PMC5619845

[ref2] Koch M. H. J.; Vachette P.; Svergun D. I. Small-angle scattering: a view on the properties, structures and structural changes of biological macromolecules in solution. Q. Rev. Biophys. 2003, 36 (2), 147–227. 10.1017/S0033583503003871. 14686102

[ref3] Kikhney A. G.; Svergun D. I. A practical guide to small angle X-ray scattering (SAXS) of flexible and intrinsically disordered proteins. FEBS Lett. 2015, 589 (19 Pt A), 2570–2577. From NLM. 10.1016/j.febslet.2015.08.027. 26320411

[ref4] Naudi-Fabra S.; Tengo M.; Jensen M. R.; Blackledge M.; Milles S. Quantitative Description of Intrinsically Disordered Proteins Using Single-Molecule FRET, NMR, and SAXS. J. Am. Chem. Soc. 2021, 143 (48), 20109–20121. 10.1021/jacs.1c06264. 34817999 PMC8662727

[ref5] Stelzl L. S.; Pietrek L. M.; Holla A.; Oroz J.; Sikora M.; Köfinger J.; Schuler B.; Zweckstetter M.; Hummer G. Global Structure of the Intrinsically Disordered Protein Tau Emerges from Its Local Structure. JACS Au 2022, 2 (3), 673–686. 10.1021/jacsau.1c00536. 35373198 PMC8970000

[ref6] Aznauryan M.; Delgado L.; Soranno A.; Nettels D.; Huang J.-r.; Labhardt A. M.; Grzesiek S.; Schuler B. Comprehensive structural and dynamical view of an unfolded protein from the combination of single-molecule FRET, NMR, and SAXS. Proc. Natl. Acad. Sci. U.S.A. 2016, 113 (37), E5389–E5398. 10.1073/pnas.1607193113. 27566405 PMC5027429

[ref7] Hub J. S. Interpreting solution X-ray scattering data using molecular simulations. Curr. Opin. Struct. Biol. 2018, 49, 18–26. 10.1016/j.sbi.2017.11.002. 29172147

[ref8] Cromer D. T.; Waber J. T. Scattering factors computed from relativistic Dirac-Slater wave functions. Acta Crystallogr. 1965, 18 (1), 104–109. 10.1107/S0365110X6500018X.

[ref9] Brown P. J.; Fox A. G.; Maslen E. N.; O’Keefe M. A.; Willis B. T. M. Intensity of Diffracted Intensities. In International Tables for Crystallography; International Union of Crystallography, 2006; pp 554–595.

[ref10] Fraser R. D. B.; MacRae T. P.; Suzuki E. An improved method for calculating the contribution of solvent to the X-ray diffraction pattern of biological molecules. J. Appl. Crystallogr. 1978, 11 (6), 693–694. 10.1107/S0021889878014296.

[ref11] Feigin L. A.; Svergun D. I. Structure Analysis by Small-Angle X-ray and Neutron Scattering; Springer Science & Business Media, 2013.

[ref12] Sears V. F. Neutron scattering lengths and cross sections. Neutron News 1992, 3 (3), 26–37. 10.1080/10448639208218770.

[ref13] Heller W. T. Small-angle neutron scattering and contrast variation: a powerful combination for studying biological structures. Acta Crystallogr., Sect. D: Biol. Crystallogr. 2010, 66 (Pt 11), 1213–1217. 10.1107/S0907444910017658. 21041939

[ref14] Svergun D. I.; Richard S.; Koch M. H.; Sayers Z.; Kuprin S.; Zaccai G. Protein hydration in solution: experimental observation by x-ray and neutron scattering. Proc. Natl. Acad. Sci. U.S.A. 1998, 95 (5), 2267–2272. 10.1073/pnas.95.5.2267. 9482874 PMC19315

[ref15] Perkins S. J. X-ray and neutron scattering analyses of hydration shells: a molecular interpretation based on sequence predictions and modelling fits. Biophys. Chem. 2001, 93 (2–3), 129–139. 10.1016/S0301-4622(01)00216-2. 11804721

[ref16] Linse J.-B.; Jochen S. H. Scrutinizing the protein hydration shell from molecular dynamics simulations against consensus small-angle scattering data. bioRxiv 2023, 10.1101/2023.06.13.544709. PMC10716392 38086909

[ref17] Merzel F.; Smith J. C. Is the first hydration shell of lysozyme of higher density than bulk water?. Proc. Natl. Acad. Sci. U.S.A. 2002, 99 (8), 5378–5383. 10.1073/pnas.082335099. 11959992 PMC122777

[ref18] Chen P.-C.; Hub J. S. Validating solution ensembles from molecular dynamics simulation by wide-angle X-ray scattering data. Biophys. J. 2014, 107 (2), 435–447. 10.1016/j.bpj.2014.06.006. 25028885 PMC4104061

[ref19] Knight C. J.; Hub J. S. WAXSiS: a web server for the calculation of SAXS/WAXS curves based on explicit-solvent molecular dynamics. Nucleic Acids Res. 2015, 43 (W1), W225–230. 10.1093/nar/gkv309. 25855813 PMC4489308

[ref20] Köfinger J.; Hummer G. Atomic-resolution structural information from scattering experiments on macromolecules in solution. Phys. Rev. E 2013, 87 (5), 052712 10.1103/PhysRevE.87.052712. 23767571

[ref21] Franke D.; Petoukhov M. V.; Konarev P. V.; Panjkovich A.; Tuukkanen A.; Mertens H. D. T.; Kikhney A. G.; Hajizadeh N. R.; Franklin J. M.; Jeffries C. M.; Svergun D. I. ATSAS 2.8: a comprehensive data analysis suite for small-angle scattering from macromolecular solutions. J. Appl. Crystallogr. 2017, 50 (Pt 4), 1212–1225. 10.1107/S1600576717007786. 28808438 PMC5541357

[ref22] Schneidman-Duhovny D.; Hammel M.; Sali A. FoXS: a web server for rapid computation and fitting of SAXS profiles. Nucleic Acids Res. 2010, 38 (Web Server issue), W540–544. 10.1093/nar/gkq461. 20507903 PMC2896111

[ref23] Grudinin S.; Garkavenko M.; Kazennov A. Pepsi-SAXS: an adaptive method for rapid and accurate computation of small-angle X-ray scattering profiles. Acta Crystallogr., Sect. D: Struct. Biol. 2017, 73 (Pt 5), 449–464. 10.1107/S2059798317005745. 28471369

[ref24] Izadi S.; Onufriev A. V. Accuracy limit of rigid 3-point water models. J. Chem. Phys. 2016, 145 (7), 074501 10.1063/1.4960175. 27544113 PMC4991989

[ref25] Conde M. M.; Gonzalez M. A.; Abascal J. L. F.; Vega C. Determining the phase diagram of water from direct coexistence simulations: the phase diagram of the TIP4P/2005 model revisited. J. Chem. Phys. 2013, 139 (15), 154505 10.1063/1.4824627. 24160525

[ref26] Bernetti M.; Bussi G. Comparing state-of-the-art approaches to back-calculate SAXS spectra from atomistic molecular dynamics simulations. Eur. Phys. J. B 2021, 94 (9), 180 10.1140/epjb/s10051-021-00186-9.

[ref27] Svergun D.; Barberato C.; Koch M. H. J. CRYSOL– a program to evaluate X-ray solution scattering of biological macromolecules from atomic coordinates. J. Appl. Crystallogr. 1995, 28 (6), 768–773. 10.1107/S0021889895007047.

[ref28] Yang S.; Park S.; Makowski L.; Roux B. A Rapid Coarse Residue-Based Computational Method for X-Ray Solution Scattering Characterization of Protein Folds and Multiple Conformational States of Large Protein Complexes. Biophys. J. 2009, 96 (11), 4449–4463. 10.1016/j.bpj.2009.03.036. 19486669 PMC2711486

[ref29] Ravikumar K. M.; Huang W.; Yang S. Fast-SAXS-pro: A unified approach to computing SAXS profiles of DNA, RNA, protein, and their complexes. J. Chem. Phys. 2013, 138 (2), 183a 10.1063/1.4774148. PMC5942439 23320673

[ref30] Stovgaard K.; Andreetta C.; Ferkinghoff-Borg J.; Hamelryck T. Calculation of accurate small angle X-ray scattering curves from coarse-grained protein models. BMC Bioinf. 2010, 11 (1), 429 10.1186/1471-2105-11-429. PMC2931518 20718956

[ref31] Zheng W.; Tekpinar M. Accurate Flexible Fitting of High-Resolution Protein Structures to Small-Angle X-Ray Scattering Data Using a Coarse-Grained Model with Implicit Hydration Shell. Biophys. J. 2011, 101 (12), 2981–2991. 10.1016/j.bpj.2011.11.003. 22208197 PMC3244063

[ref32] Niebling S.; Bjorling A.; Westenhoff S. MARTINI bead form factors for the analysis of time-resolved X-ray scattering of proteins. J. Appl. Crystallogr. 2014, 47 (4), 1190–1198. 10.1107/S1600576714009959. 25242909 PMC4119947

[ref33] Marrink S. J.; Tieleman D. P. Perspective on the Martini model. Chem. Soc. Rev. 2013, 42 (16), 6801–6822. 10.1039/c3cs60093a. 23708257

[ref34] Paissoni C.; Jussupow A.; Camilloni C. Martini bead form factors for nucleic acids and their application in the refinement of protein-nucleic acid complexes against SAXS data. J. Appl. Crystallogr. 2019, 52 (2), 394–402. 10.1107/S1600576719002450.

[ref35] Jussupow A.; Messias A. C.; Stehle R.; Geerlof A.; Solbak S. M. Ø.; Paissoni C.; Bach A.; Sattler M.; Camilloni C. The dynamics of linear polyubiquitin. Sci. Adv. 2020, 6 (42), eabc3786 10.1126/sciadv.abc3786. 33055165 PMC7556843

[ref36] Paissoni C.; Jussupow A.; Camilloni C. Determination of Protein Structural Ensembles by Hybrid-Resolution SAXS Restrained Molecular Dynamics. J. Chem. Theory Comput. 2020, 16 (4), 2825–2834. 10.1021/acs.jctc.9b01181. 32119546 PMC7997378

[ref37] Saad D.; Paissoni C.; Chaves-Sanjuan A.; Nardini M.; Mantovani R.; Gnesutta N.; Camilloni C. High Conformational Flexibility of the E2F1/DP1/DNA Complex. J. Mol. Biol. 2021, 433 (18), 167119 10.1016/j.jmb.2021.167119. 34181981

[ref38] Paissoni C.; Camilloni C. How to Determine Accurate Conformational Ensembles by Metadynamics Metainference: A Chignolin Study Case. Front. Mol. Biosci. 2021, 8, 694130 10.3389/fmolb.2021.694130. 34124166 PMC8187852

[ref39] Ahmed M. C.; Skaanning L. K.; Jussupow A.; Newcombe E. A.; Kragelund B. B.; Camilloni C.; Langkilde A. E.; Lindorff-Larsen K. Refinement of α-Synuclein Ensembles Against SAXS Data: Comparison of Force Fields and Methods. Front. Mol. Biosci. 2021, 8, 654333 10.3389/fmolb.2021.654333. 33968988 PMC8100456

[ref40] Nag S.; Larsson M.; Robinson R. C.; Burtnick L. D. Gelsolin: The tail of a molecular gymnast. Cytoskeleton 2013, 70 (7), 360–384. 10.1002/cm.21117. 23749648

[ref41] Yin H. L.; Stossel T. P. Control of cytoplasmic actin gel–sol transformation by gelsolin, a calcium-dependent regulatory protein. Nature 1979, 281 (5732), 583–586. 10.1038/281583a0. 492320

[ref42] Sun H. Q.; Yamamoto M.; Mejillano M.; Yin H. L. Gelsolin, a multifunctional actin regulatory protein. J. Biol. Chem. 1999, 274 (47), 33179–33182. From NLM. 10.1074/jbc.274.47.33179. 10559185

[ref43] Piktel E.; Levental I.; Durnaś B.; Janmey P. A.; Bucki R. Plasma Gelsolin: Indicator of Inflammation and Its Potential as a Diagnostic Tool and Therapeutic Target. Int. J. Mol. Sci. 2018, 19 (9), 2516 10.3390/ijms19092516. 30149613 PMC6164782

[ref44] Solomon J. P.; Page L. J.; Balch W. E.; Kelly J. W. Gelsolin amyloidosis: genetics, biochemistry, pathology and possible strategies for therapeutic intervention. Crit. Rev. Biochem. Mol. Biol. 2012, 47 (3), 282–296. 10.3109/10409238.2012.661401. 22360545 PMC3337338

[ref45] Li G. H.; Arora P. D.; Chen Y.; McCulloch C. A.; Liu P. Multifunctional roles of gelsolin in health and diseases. Med. Res. Rev. 2012, 32 (5), 999–1025. 10.1002/med.20231. 22886630

[ref46] Ashish; Paine M. S.; Perryman P. B.; Yang L.; Yin H. L.; Krueger J. K. Global Structure Changes Associated with Ca2+ Activation of Full-length Human Plasma Gelsolin*. J. Biol. Chem. 2007, 282 (35), 25884–25892. 10.1074/jbc.M702446200. 17604278

[ref47] Nag S.; Ma Q.; Wang H.; Chumnarnsilpa S.; Lee W. L.; Larsson M.; Kannan B.; Hernandez-Valladares M.; Burtnick L. D.; Robinson R. C. Ca^2+^ binding by domain 2 plays a critical role in the activation and stabilization of gelsolin. Proc. Natl. Acad. Sci. U.S.A. 2009, 106 (33), 13713–13718. 10.1073/pnas.0812374106. 19666512 PMC2720848

[ref48] Zorgati H.; Larsson M.; Ren W.; Sim A. Y. L.; Gettemans J.; Grimes J. M.; Li W.; Robinson R. C. The role of gelsolin domain 3 in familial amyloidosis (Finnish type). Proc. Natl. Acad. Sci. U.S.A. 2019, 116 (28), 13958–13963. 10.1073/pnas.1902189116. 31243148 PMC6628662

[ref49] de Rosa M.; Barbiroli A.; Bonì F.; Scalone E.; Mattioni D.; Vanoni M. A.; Patrone M.; Bollati M.; Mastrangelo E.; Giorgino T.; Milani M. The structure of N184K amyloidogenic variant of gelsolin highlights the role of the H-bond network for protein stability and aggregation properties. Eur. Biophys. J. 2020, 49 (1), 11–19. 10.1007/s00249-019-01409-9. 31724080

[ref50] Bollati M.; Diomede L.; Giorgino T.; Natale C.; Fagnani E.; Boniardi I.; Barbiroli A.; Alemani R.; Beeg M.; Gobbi M.; et al. A novel hotspot of gelsolin instability triggers an alternative mechanism of amyloid aggregation. Comput. Struct. Biotechnol. J. 2021, 19, 6355–6365. 10.1016/j.csbj.2021.11.025. 34938411 PMC8649582

[ref51] Bonomi M.; Camilloni C. Integrative structural and dynamical biology with PLUMED-ISDB. Bioinformatics 2017, 33 (24), 3999–4000. 10.1093/bioinformatics/btx529. 28961689

[ref52] Tribello G. A.; Bonomi M.; Branduardi D.; Camilloni C.; Bussi G. PLUMED 2: New feathers for an old bird. Comput. Phys. Commun. 2014, 185 (2), 604–613. 10.1016/j.cpc.2013.09.018.

[ref53] Bonomi M.; Bussi G.; Camilloni C.; Tribello G. A.; Banáš P.; Barducci A.; Bernetti M.; Bolhuis P. G.; Bottaro S.; Branduardi D.; et al. Promoting transparency and reproducibility in enhanced molecular simulations. Nat. Methods 2019, 16 (8), 670–673. 10.1038/s41592-019-0506-8. 31363226

[ref54] Weiser J.; Shenkin P. S.; Still W. C. Approximate atomic surfaces from linear combinations of pairwise overlaps (LCPO). J. Comput. Chem. 1999, 20 (2), 217–230. 10.1002/(SICI)1096-987X(19990130)20:2<217::AID-JCC4>3.0.CO;2-A.

[ref55] Šponer J.; Bussi G.; Krepl M.; Banáš P.; Bottaro S.; Cunha R. A.; Gil-Ley A.; Pinamonti G.; Poblete S.; Jurečka P.; et al. RNA Structural Dynamics As Captured by Molecular Simulations: A Comprehensive Overview. Chem. Rev. 2018, 118 (8), 4177–4338. 10.1021/acs.chemrev.7b00427. 29297679 PMC5920944

[ref56] Svozil D.; Kalina J.; Omelka M.; Schneider B. DNA conformations and their sequence preferences. Nucleic Acids Res. 2008, 36 (11), 3690–3706. 10.1093/nar/gkn260. 18477633 PMC2441783

[ref57] Bernauer J.; Huang X.; Sim A. Y. L.; Levitt M. Fully differentiable coarse-grained and all-atom knowledge-based potentials for RNA structure evaluation. RNA 2011, 17 (6), 1066–1075. 10.1261/rna.2543711. 21521828 PMC3096039

[ref58] Jumper J.; Evans R.; Pritzel A.; Green T.; Figurnov M.; Ronneberger O.; Tunyasuvunakool K.; Bates R.; Žídek A.; Potapenko A.; et al. Highly accurate protein structure prediction with AlphaFold. Nature 2021, 596 (7873), 583–589. 10.1038/s41586-021-03819-2. 34265844 PMC8371605

[ref59] Madhavi Sastry G.; Adzhigirey M.; Day T.; Annabhimoju R.; Sherman W. Protein and ligand preparation: parameters, protocols, and influence on virtual screening enrichments. J. Comput.-Aided Mol. Des. 2013, 27 (3), 221–234. 10.1007/s10822-013-9644-8. 23579614

[ref60] Piana S.; Donchev A. G.; Robustelli P.; Shaw D. E. Water Dispersion Interactions Strongly Influence Simulated Structural Properties of Disordered Protein States. J. Phys. Chem. B 2015, 119 (16), 5113–5123. 10.1021/jp508971m. 25764013

[ref61] Bonomi M.; Camilloni C.; Cavalli A.; Vendruscolo M. Metainference: A Bayesian inference method for heterogeneous systems. Sci.Adv. 2016, 2 (1), e1501177 10.1126/sciadv.1501177. 26844300 PMC4737209

[ref62] Maier J. A.; Martinez C.; Kasavajhala K.; Wickstrom L.; Hauser K. E.; Simmerling C. ff14SB: Improving the Accuracy of Protein Side Chain and Backbone Parameters from ff99SB. J. Chem. Theory Comput. 2015, 11 (8), 3696–3713. 10.1021/acs.jctc.5b00255. 26574453 PMC4821407

[ref63] Ivani I.; Dans P. D.; Noy A.; Pérez A.; Faustino I.; Hospital A.; Walther J.; Andrio P.; Goñi R.; Balaceanu A.; et al. Parmbsc1: a refined force field for DNA simulations. Nat. Methods 2016, 13 (1), 55–58. 10.1038/nmeth.3658. 26569599 PMC4700514

[ref64] Jorgensen W. L.; Chandrasekhar J.; Madura J. D.; Impey R. W.; Klein M. L. Comparison of simple potential functions for simulating liquid water. J. Chem. Phys. 1983, 79 (2), 926–935. 10.1063/1.445869.

[ref65] Kooshapur H.; Choudhury N. R.; Simon B.; Mühlbauer M.; Jussupow A.; Fernandez N.; Jones A. N.; Dallmann A.; Gabel F.; Camilloni C.; et al. Structural basis for terminal loop recognition and stimulation of pri-miRNA-18a processing by hnRNP A1. Nat. Commun. 2018, 9 (1), 2479 10.1038/s41467-018-04871-9. 29946118 PMC6018666

[ref66] Abraham M. J.; Murtola T.; Schulz R.; Páll S.; Smith J. C.; Hess B.; Lindahl E. GROMACS: High performance molecular simulations through multi-level parallelism from laptops to supercomputers. SoftwareX 2015, 1–2, 19–25. 10.1016/j.softx.2015.06.001.

[ref67] Hunter J. D. Matplotlib: A 2D Graphics Environment. Comput. Sci. Eng. 2007, 9 (3), 90–95. 10.1109/MCSE.2007.55.

[ref68] Humphrey W.; Dalke A.; Schulten K. VMD: Visual molecular dynamics. J. Mol. Graphics 1996, 14 (1), 33–38. 10.1016/0263-7855(96)00018-5. 8744570

[ref69] Schrödinger, L. L. C. The PyMOL Molecular Graphics System, Version 2.6.

[ref70] Ballabio F.; Capelli R.; Camilloni C. Supporting data for: ″An accurate and efficient SAXS/SANS implementation including solvation layer effects suitable for restrained Molecular Dynamics simulations.”. Zenodo: 2023. 10.1021/acs.jctc.3c00864 PMC10687869 37923304

[ref71] Giorgino T.; Mattioni D.; Hassan A.; Milani M.; Mastrangelo E.; Barbiroli A.; Verhelle A.; Gettemans J.; Barzago M. M.; Diomede L.; de Rosa M. Nanobody interaction unveils structure, dynamics and proteotoxicity of the Finnish-type amyloidogenic gelsolin variant. Biochim. Biophys. Acta, Mol. Basis Dis. 2019, 1865 (3), 648–660. 10.1016/j.bbadis.2019.01.010. 30625383

[ref72] Cowieson N. P.; Edwards-Gayle C. J. C.; Inoue K.; Khunti N. S.; Doutch J.; Williams E.; Daniels S.; Preece G.; Krumpa N. A.; Sutter J. P.; et al. Beamline B21: high-throughput small-angle X-ray scattering at Diamond Light Source. J. Synchrotron Radiat. 2020, 27 (5), 1438–1446. 10.1107/S1600577520009960. 32876621 PMC7467336

[ref73] Valentini E.; Kikhney A. G.; Previtali G.; Jeffries C. M.; Svergun D. I. SASBDB, a repository for biological small-angle scattering data. Nucleic Acids Res. 2015, 43 (D1), D357–D363. 10.1093/nar/gku1047. 25352555 PMC4383894

[ref74] Arsiccio A.; Shea J.-E. Protein Cold Denaturation in Implicit Solvent Simulations: A Transfer Free Energy Approach. J. Phys. Chem. B 2021, 125 (20), 5222–5232. 10.1021/acs.jpcb.1c01694. 33988995

[ref75] Eisenhaber F.; Lijnzaad P.; Argos P.; Sander C.; Scharf M. The double cubic lattice method: Efficient approaches to numerical integration of surface area and volume and to dot surface contouring of molecular assemblies. J. Comput. Chem. 1995, 16 (3), 273–284. 10.1002/jcc.540160303.

[ref76] Takeda S.; Fujiwara I.; Sugimoto Y.; Oda T.; Narita A.; Maéda Y. Novel inter-domain Ca2+-binding site in the gelsolin superfamily protein fragmin. J. Muscle Res. Cell Motil. 2020, 41 (1), 153–162. 10.1007/s10974-019-09571-5. 31863323

[ref77] Bollati M.; Scalone E.; Bonì F.; Mastrangelo E.; Giorgino T.; Milani M.; de Rosa M. High-resolution crystal structure of gelsolin domain 2 in complex with the physiological calcium ion. Biochem. Biophys. Res. Commun. 2019, 518 (1), 94–99. 10.1016/j.bbrc.2019.08.013. 31416615

[ref78] Vorobiev S.; Strokopytov B.; Drubin D. G.; Frieden C.; Ono S.; Condeelis J.; Rubenstein P. A.; Almo S. C. The structure of nonvertebrate actin: Implications for the ATP hydrolytic mechanism. Proc. Natl. Acad. Sci. U.S.A. 2003, 100 (10), 5760–5765. 10.1073/pnas.0832273100. 12732734 PMC156274

[ref79] Bonomi M.; Heller G. T.; Camilloni C.; Vendruscolo M. Principles of protein structural ensemble determination. Curr. Opin. Struct. Biol. 2017, 42, 106–116. From NLM. 10.1016/j.sbi.2016.12.004. 28063280

[ref80] Orioli S.; Larsen A. H.; Bottaro S.; Lindorff-Larsen K. How to learn from inconsistencies: Integrating molecular simulations with experimental data. Prog. Mol. Biol. Transl. Sci. 2020, 170, 123–176. From NLM. 10.1016/bs.pmbts.2019.12.006. 32145944

[ref81] Habeck M. Bayesian methods in integrative structure modeling. Biol. Chem. 2023, 404, 741 10.1515/hsz-2023-0145. 37505205

[ref82] Hummer G.; Köfinger J. Bayesian ensemble refinement by replica simulations and reweighting. J. Chem. Phys. 2015, 143 (24), 243150 10.1063/1.4937786. 26723635

[ref83] Rangan R.; Bonomi M.; Heller G. T.; Cesari A.; Bussi G.; Vendruscolo M. Determination of Structural Ensembles of Proteins: Restraining vs Reweighting. J. Chem. Theory Comput. 2018, 14 (12), 6632–6641. 10.1021/acs.jctc.8b00738. 30428663

[ref84] Cavalli A.; Camilloni C.; Vendruscolo M. Molecular dynamics simulations with replica-averaged structural restraints generate structural ensembles according to the maximum entropy principle. J. Chem. Phys. 2013, 138 (9), 094112 10.1063/1.4793625. 23485282

[ref85] Cesari A.; Gil-Ley A.; Bussi G. Combining Simulations and Solution Experiments as a Paradigm for RNA Force Field Refinement. J. Chem. Theory Comput. 2016, 12 (12), 6192–6200. 10.1021/acs.jctc.6b00944. 27951677

[ref86] Capelli R.; Tiana G.; Camilloni C. An implementation of the maximum-caliber principle by replica-averaged time-resolved restrained simulations. J. Chem. Phys. 2018, 148 (18), 184114 10.1063/1.5030339. 29764124

[ref87] Laio A.; Parrinello M. Escaping free-energy minima. Proc. Natl. Acad. Sci. U.S.A. 2002, 99 (20), 12562–12566. 10.1073/pnas.202427399. 12271136 PMC130499

[ref88] Torrie G. M.; Valleau J. P. Nonphysical sampling distributions in Monte Carlo free-energy estimation: Umbrella sampling. J. Comput. Phys. 1977, 23 (2), 187–199. 10.1016/0021-9991(77)90121-8.

