# Supplementary material for: Accurate and Efficient SAXS/SANS Implementation Including Solvation Layer Effects Suitable for Molecular Simulations
Source: J Chem Theory Comput. 2023 Nov 3;19(22):8401–13. doi: 10.1021/acs.jctc.3c00864 (PMC10687869; doi:10.1021/acs.jctc.3c00864)
Supplement: Supplementary file 1 — ct3c00864_si_001.pdf [file ct3c00864_si_001.pdf]

Supporting Information for: An Accurate and Efficient SAXS/SANS Implementation Including Solvation Layer Effects Suitable for Molecular Simulations.

Federico Ballabio[1], Cristina Paissoni[1], Michela Bollati[1,2], Matteo de Rosa[1,2,*], Riccardo Capelli[1,*], and Carlo Camilloni[1*].

[1]Dipartimento di Bioscienze, Università degli Studi di Milano, via Celoria 26, 20133 Milano, Italy

[2]Istituto di Biofisica, Consiglio Nazionale delle Ricerche (IBF-CNR), via Alfonso Corti 12, 20133 Milano, Italy

Type	PDB Codes
RNA	157D, 1CSL, 1D4R, 1DQF, 1DUH, 1DUQ, 1F1T, 1F27, 1FIR, 1G2J, 1I9V, 1I9X, 1J9H, 1JZV, 1K9W, 1KD5, 1KFO, 1KH6, 1KXK, 1L2X, 1L3Z, 1MHK, 1MME, 1MSY, 1NBS, 1NUJ, 1P79, 1Q93, 1QBP, 1SA9, 1SDR, 1T0D, 1T0E, 1U9S, 1X8W, 1X9C, 1X9K, 1XJR, 1Y0Q, 1YFG, 1YKQ, 1YZD, 1Z43, 1Z58, 205D, 255D, 259D, 280D, 2A0P, 2A2E, 2A64, 2AO5, 2B8R, 2G3S, 2G91, 2H0S, 2NOK, 2OE6, 2TRA, 333D, 353D, 357D, 361D, 377D, 387D, 397D, 402D, 405D, 406D, 409D, 413D, 433D, 434D, 438D, 472D.
DNA (A-form)	118D, 137D, 138D, 160D, 1D78, 1D79, 1DNZ, 1KGK, 1M77, 1MA8, 1MLX, 1NZG, 1VJ4, 1VT5, 1VTB, 1XJX, 1Z7I, 1ZEX, 1ZEY, 1ZF1, 1ZF6, 1ZF8, 1ZF9, 1ZFA, 243D, 260D, 295D, 2D94, 317D, 338D, 344D, 345D, 348D, 349D, 368D, 369D, 370D, 371D, 395D, 396D, 399D, 414D, 440D, 9DNA.
DNA (B-form)	122D, 123D, 158D, 183D, 196D, 1BD1, 1BNA, 1CW9, 1D23, 1D3R, 1D49, 1D56, 1D8G, 1D8X, 1DOU, 1DPN, 1EDR, 1EHV, 1EN3, 1EN8, 1EN9, 1ENE, 1ENN, 1FQ2, 1G75, 1I3T, 1IKK, 1J8L, 1JGR, 1L4J, 1L6B, 1M6G, 1N1O, 1NVN, 1NVY, 1P4Y, 1P54, 1S23, 1S2R, 1SGS, 1SK5, 1UB8, 1VE8, 1ZF0, 1ZF3, 1ZF4, 1ZF5, 1ZF7, 1ZFB, 1ZFF, 1ZFG, 232D, 251D, 2D25, 307D, 355D, 3DNB, 403D, 423D, 428D, 431D, 436D, 454D, 455D, 456D, 460D,463D, 476D, 477D, 5DNB, 9BNA.
DNA (Z-form)	131D, 145D, 181D, 1D48, 1D53, 1DA2, 1DCG, 1DJ6, 1DNF, 1I0T, 1ICK, 1JES, 1LJX, 1OMK, 1VTT, 1VTW, 1XA2, 1XAM, 1ZNA, 210D, 211D, 242D, 292D, 293D, 2DCG, 313D, 314D, 331D, 336D, 351D, 362D, 400D,417D.
DNA (Quadruplexes	184D, 190D, 191D, 1BQJ, 1CN0, 1JPQ, 1L1H, 1MF5, 1O0K, 1QYK, 1QYL, 1V3N, 1V3O, 1V3P, 200D, 241D, 244D, 284D, 352D.

Table S1. List of PDB files used to compute the 3B parameters. The underlined 43 codes for RNA and 120 for DNA indicate the structures belonging to the initial training set. The remaining 32 RNA structures and 47 DNA structures were used as the validation set. The final parameters were calculated from the full set of 242 PDB structures.

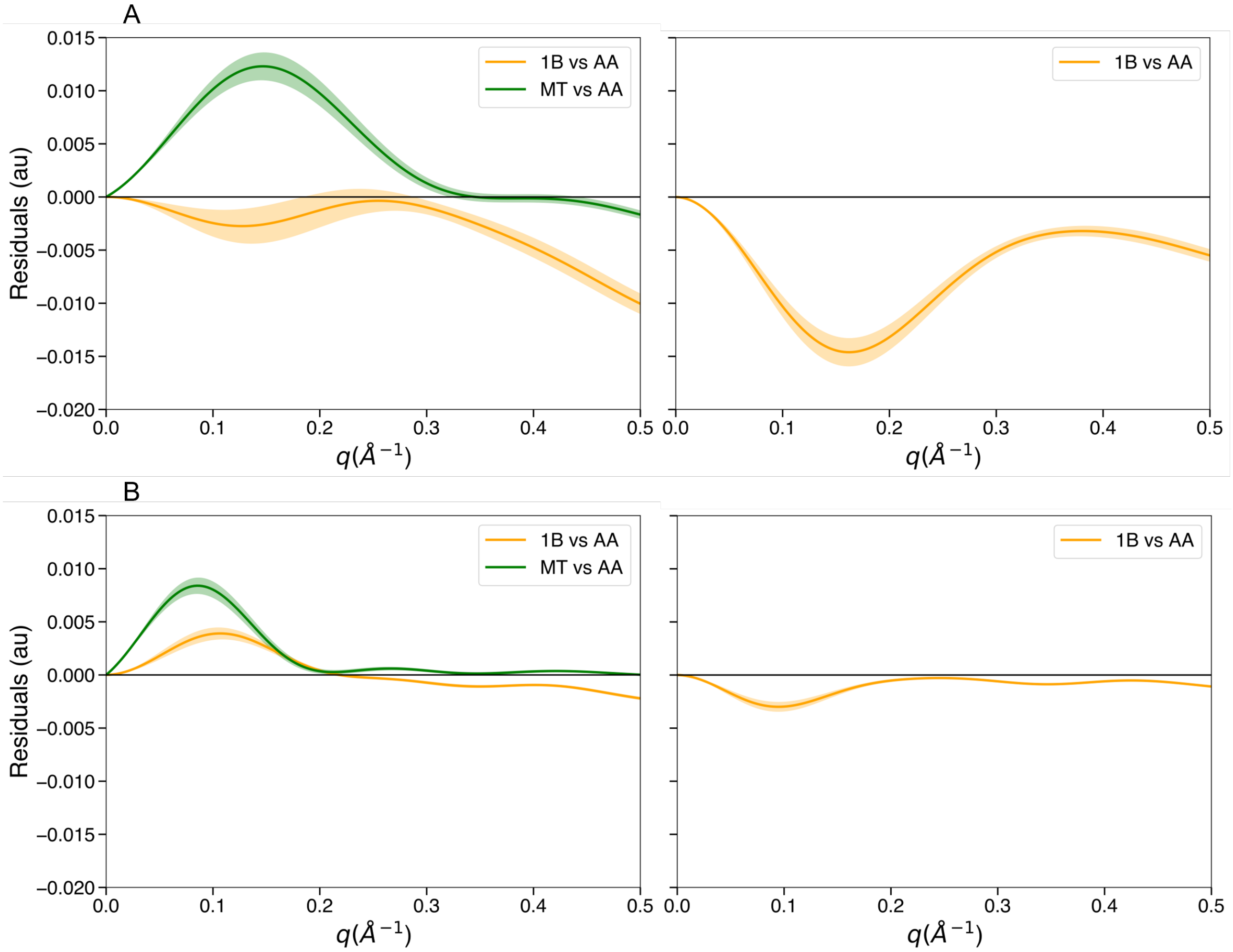

Figure S1. Accuracy evaluation of coarse-grained mappings in the calculation of scattering intensities. The SAS profile of each frame from MD trajectories was calculated with coarse-grained mappings and at AA resolution, for 201 q values ranging from $1{\cdot}10^{-10}$ Å^{-1} to 0.5 Å^{-1}. A) Left panel: average and standard deviation on 6,502 B1 frames of the SAXS residuals between MT and AA (green), and between 1B and AA (orange). Right panel: average and standard deviation of SANS residual between 1B and AA (orange). B) Left panel: average and standard deviation on 9,622 GFP frames of the SAXS residuals between MT and AA (green), and between 1B and AA (orange). Right panel: average and standard deviation of SANS residual between 1B and AA (orange).

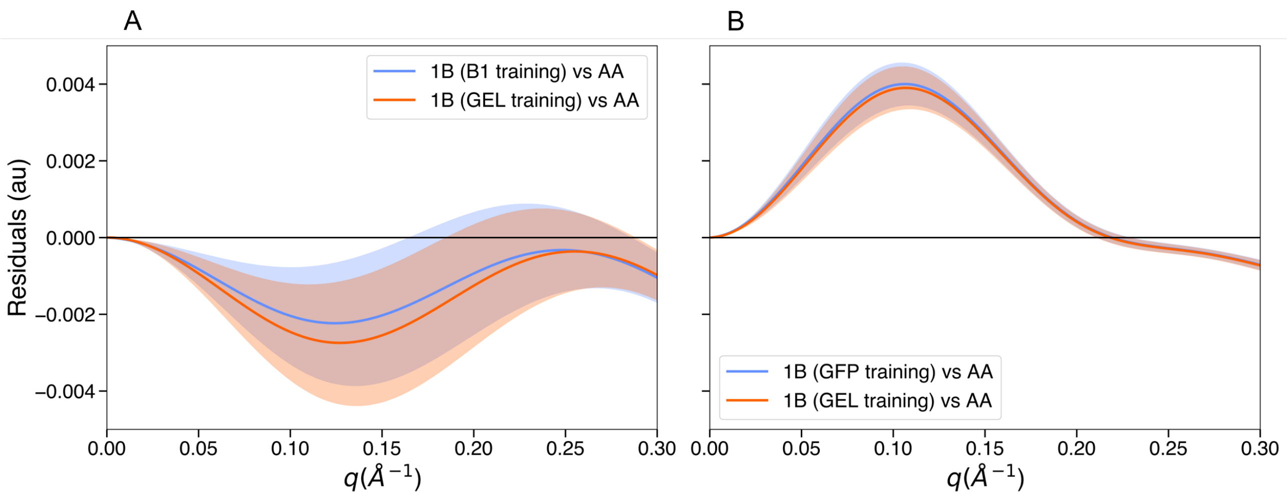

Figure S2. Transferability assessment of 1B parameters. The SAXS profile of each frame from B1 and GFP MD trajectories was calculated with 1B mapping employing B1 and GFP parameters, respectively, with 1B mapping and GSN parameters, and at AA resolution, for 201 q values ranging from $1{\cdot}10^{-10}$ Å^{-1} to 0.3 Å^{-1} A) Average and standard deviation on 6,502 B1 frames of the SAXS residuals between 1B with B1 parameters and AA (blue), and between 1B with GSN parameters and AA (red). B) Average and standard deviation on 9,622 GFP frames of the SAXS residuals between 1B with GFP parameters and AA (blue), and between 1B with GSN parameters and AA (red).

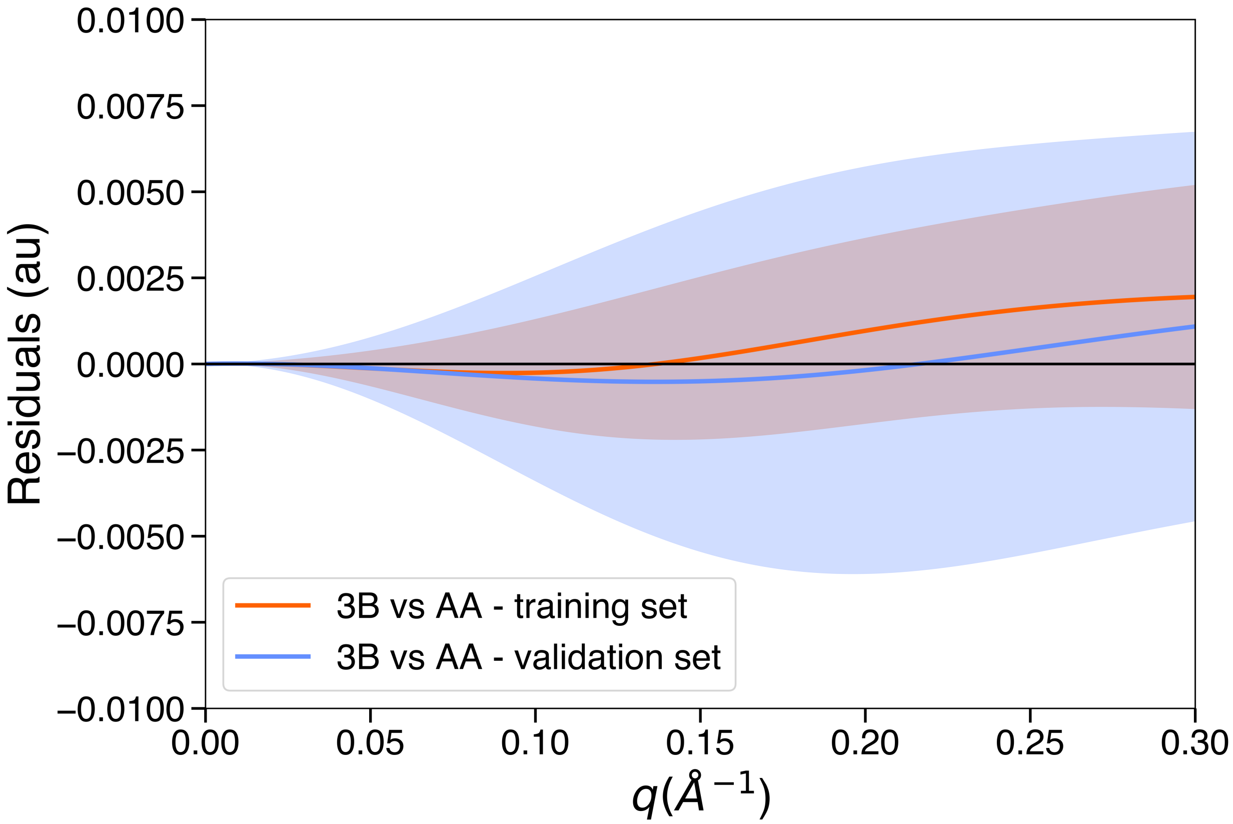

Figure S3. Transferability assessment of 3B parameters. The SAXS profile of each structure belonging to the training and to the validation sets was calculated with 3B mapping and the parameters computed from the PDB training set, and at AA resolution. The intensity was calculated for 201 *q* values ranging from $1 \cdot 10^{-10}$ Å^{-1} to 0.3 Å^{-1}. In red the average and standard deviation on 163 PDB structures (training set) of the SAXS residuals between 3B mapping and AA resolution. In blue the average and standard deviation on 79 PDB structures (validation set) of the SAXS residuals between 3B mapping and AA resolution.

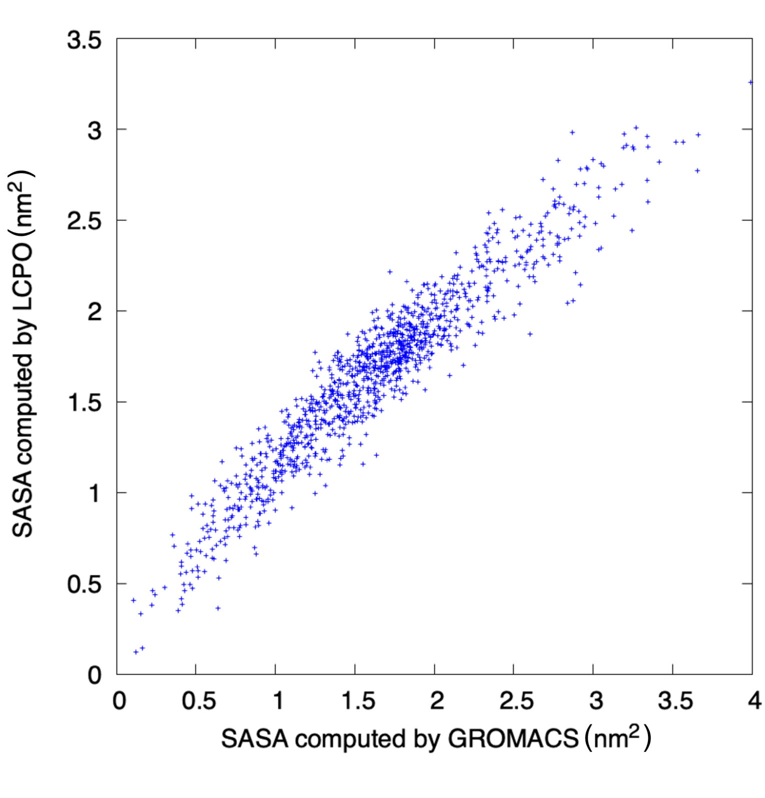

Figure S4. Comparison of SASA calculation between PLUMED LCPO and GROMACS. The SASA of each residue of a GSN frame randomly extracted from a MD trajectory was computed using the LCPO algorithm implemented in PLUMED (y-axis) and with the sasa module of GROMACS (x-axis). Each blue cross represents one residue.

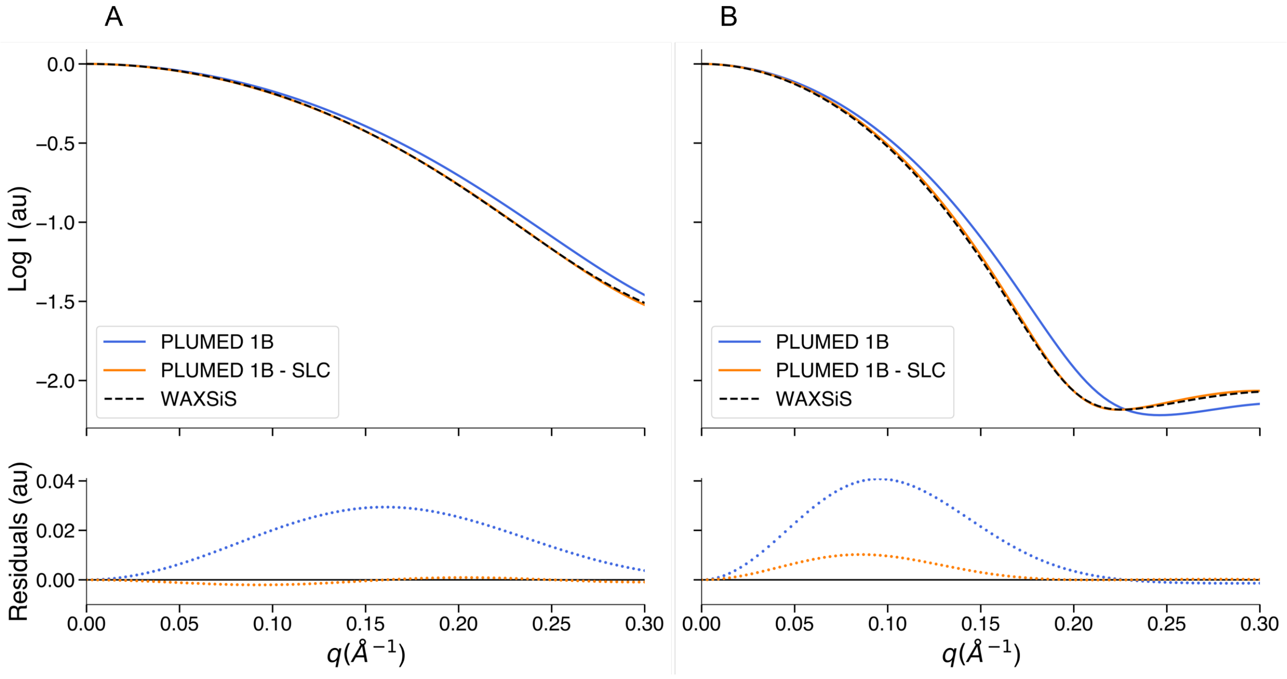

Figure S5. The solvation layer contribution in the 1B SAXS intensity calculation. A) Upper panel: base 10 logarithm of the SAXS profile of a representative, randomly selected, B1 frame calculated using 1B mapping (blue), 1B mapping with the best combination of SLC (0.09) and SC (1.2 nm^2) found for this frame (orange) and using WAXSiS (black dashed line). Bottom panel: residuals of 1B (blue) and 1B with SLC (orange) using the WAXSiS intensity as reference. B) Upper panel: base 10 logarithm of the SAXS profile of a representative, randomly selected, GFP frame calculated using 1B mapping (blue), 1B mapping with the best combination of SLC (0.08) and SC (0.7 nm^2) found for this frame (orange) and using WAXSiS (black dashed line). Bottom panel: residuals of 1B (blue) and 1B with SLC (orange) using the WAXSiS intensity as reference. All the SAXS intensities were calculated for 101 q values, up to 0.3 $Å^{-1}$.

A

MAPPING	SLC	RMSE (e-02) SC: 1.2	SC: 1.0	SC: 0.8	SC: 0.7	SC: 0.6	SC: 0.4	//
AA	//	//	//	//	//	//	//	6.5
MT	//	//	//	//	//	//	//	7.8
1B	//	//	//	//	//	//	//	7.1
1B	0.040	5.2	4.4	3.7	3.5	3.4	3.6	//
1B	0.060	4.5	3.5	2.7	2.5	2.4	2.8	//
1B	0.070	4.2	3.2	2.4	2.2	2.1	2.6	//
1B	0.080	4.0	2.9	2.2	2.1	2.0	2.4	//
1B	0.090	3.8	2.7	2.2	2.2	2.1	2.4	//
1B	0.095	3.7	2.7	2.3	2.2	2.2	2.3	//
1B	0.100	3.6	2.6	2.3	2.3	2.3	2.4	//
1B	0.110	3.5	2.6	2.5	2.6	2.6	2.4	//
1B	0.120	3.4	2.7	2.8	2.8	2.9	2.5	//

B

MAPPING	SLC	RMSE (e-02) SC: 1.2	SC: 1.0	SC: 0.8	SC: 0.7	SC: 0.6	SC: 0.4	//
AA	//	//	//	//	//	//	//	7.5
MT	//	//	//	//	//	//	//	9.0
1B	//	//	//	//	//	//	//	7.2
1B	0.040	4.7	4.3	4.0	3.9	4.0	4.2	//
1B	0.060	3.7	3.3	3.0	2.9	3.1	3.4	//
1B	0.070	3.2	2.9	2.6	2.5	2.7	3.1	//
1B	0.080	2.8	2.5	2.3	2.2	2.4	2.8	//
1B	0.090	2.5	2.3	2.0	1.9	2.2	2.6	//
1B	0.095	2.4	2.2	1.9	1.8	2.1	2.5	//
1B	0.100	2.2	2.1	1.8	1.6	2.0	2.4	//
1B	0.110	2.0	2.0	1.6	1.5	1.8	2.3	//
1B	0.120	1.9	1.9	1.5	1.4	1.8	2.2	//

C

MAPPING	SLC	RMSE (e-02) SC: 1.2	SC: 1.0	SC: 0.8	SC: 0.7	SC: 0.6	SC: 0.4	//
AA	//	//	//	//	//	//	//	7.0
MT	//	//	//	//	//	//	//	8.0
1B	//	//	//	//	//	//	//	8.1
1B	0.040	6.2	5.5	4.6	4.3	4.2	4.2	//
1B	0.060	5.4	4.5	3.6	3.3	3.3	3.6	//
1B	0.070	5.1	4.1	3.3	3.1	3.1	3.7	//
1B	0.080	4.8	3.9	3.2	3.1	3.2	4.0	//
1B	0.090	4.5	3.7	3.2	3.2	3.5	4.4	//
1B	0.095	4.4	3.6	3.3	3.3	3.7	4.6	//
1B	0.100	4.4	3.6	3.4	3.5	3.9	4.8	//
1B	0.110	4.3	3.5	3.6	3.9	4.4	5.4	//
1B	0.120	4.2	3.6	4.0	4.4	4.9	5.9	//

D

MAPPING	SLC	RMSE (e-02) SC: 1.2	SC: 1.0	SC: 0.8	SC: 0.7	SC: 0.6	SC: 0.4	//
AA	//	//	//	//	//	//	//	3.9
MT	//	//	//	//	//	//	//	4.7
3B	//	//	//	//	//	//	//	3.8
3B	0.040	3.1	2.9	2.9	2.9	3.1	3.4	//
3B	0.060	2.8	2.5	2.5	2.6	2.9	3.3	//
3B	0.070	2.7	2.4	2.3	2.5	2.8	3.1	//
3B	0.080	2.5	2.2	2.2	2.3	2.7	3.1	//
3B	0.090	2.4	2.1	2.0	2.2	2.6	3.1	//
3B	0.095	2.4	2.0	2.0	2.2	2.5	3.0	//
3B	0.100	2.3	2.0	1.9	2.1	2.6	3.0	//
3B	0.110	2.2	1.8	1.8	2.0	2.4	3.0	//
3B	0.120	2.1	1.7	1.7	1.9	2.3	2.9	//

Table S2. SLC and SC evaluation in SAXS intensity calculation. Each table shows the RMSE between the logarithm (base 10) of the SAXS intensity calculated with AA, MT, 1B/3B (with different values of SLC/SC) and the logarithm of the SAXS intensity calculated with WAXSiS, averaged for 10 equidistant frames extracted from A) GSN, B) B1, C) GFP and D) 12-*mer* RNA MD trajectories. The SC is expressed in nm^2.

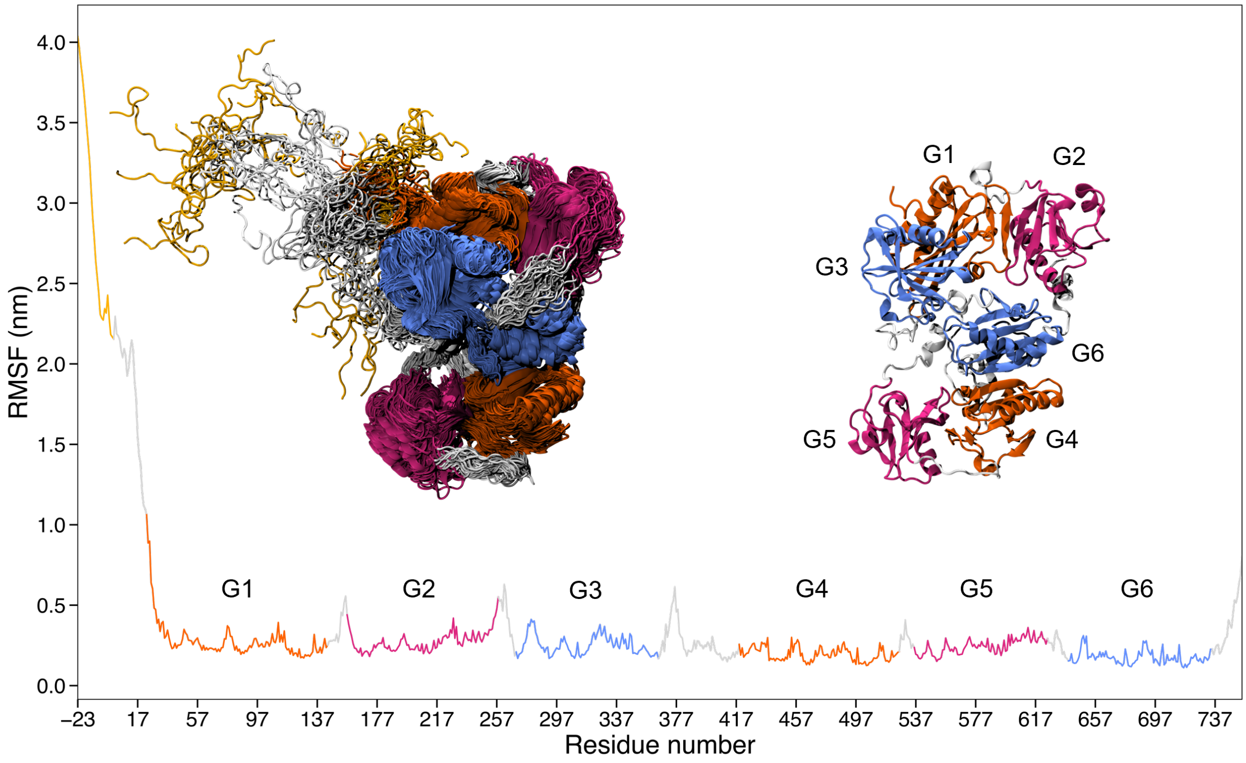

Figure S6. RMSF analysis of the GSN ensemble (without SLC). The flexibility of the protein was assessed by calculating the root-mean-square-fluctuation of all residues. The residue numbering sequence on the x-axis includes the N-terminal His_6-tag (from -23 to -1) and the full-length human plasma isoform of GSN (1 to 755). The domains sharing the highest sequence and structural similarity are shown with the same colour code: G1 and G4 in orange, G2 and G5 in purple, G3 and G6 in blue. The linkers and tails are coloured in light grey while the His_6-tag is coloured in yellow. On the left, 50 equidistant frames from the analysed trajectories are superimposed as a representative example of the conformational ensemble. The GSN structure on the right is that obtained by X-ray crystallography (PDB ID: 3FFN).

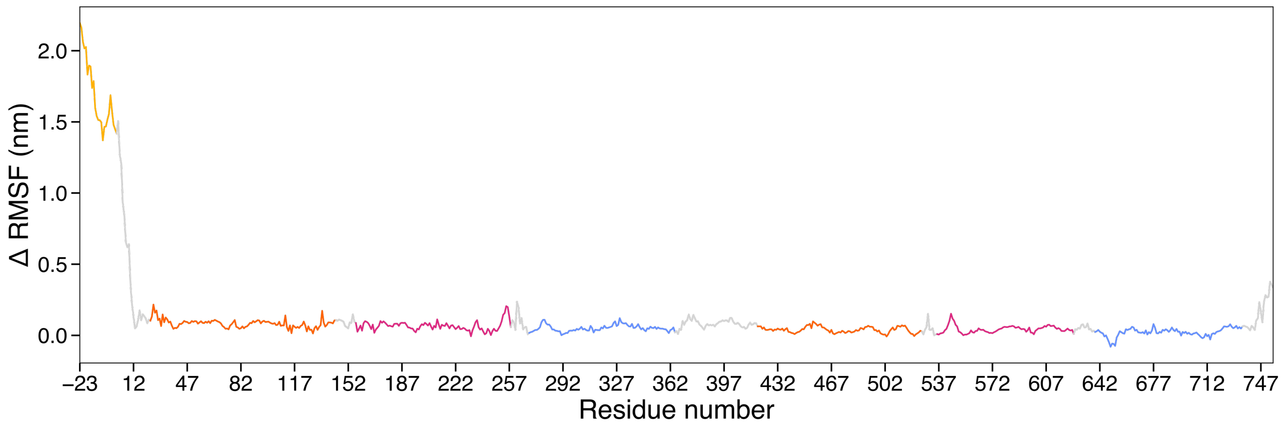

Figure S7. Difference in RMSF between the residues of the GSN ensemble obtained with SLC and the residues of the GSN ensemble obtained without SLC. The residue numbering sequence on the x-axis includes the N-terminal His6-tag (from -23 to -1) and the full-length human plasma isoform of GSN (1 to 755). The domains sharing the highest sequence and structural similarity are shown with the same colour code: G1 and G4 in orange, G2 and G5 in purple, G3 and G6 in blue. The linkers and tails are coloured in light grey while the His_6-tag is coloured in yellow.
